# Investigation of carbonation-induced microstructural changes in low-cement concrete using sustainable binders: GGBS and calcium carbonate

**DOI:** 10.1038/s41598-026-45725-5

**Published:** 2026-03-24

**Authors:** B. Narendra Kumar, Prakhash Neelamegam, Akkala Prasanna Divya Sai, Missgna Addisalem Berhe

**Affiliations:** 1 Department of Civil Engineering, VNR Vignana Jyothi Institute of Engineering &Technology, Hyderabad, Telangana India; 2 Department of Civil Engineering, School of Engineering, SR University, Warangal, Telangana 506371 India; 3 Department of Civil Engineering, Adigrat University, Adigrat, Ethiopia

**Keywords:** Calcium carbonate, Ground granulated blast furnace slag, Microstructural analysis, Nyquist plots and bode plots, Engineering, Environmental sciences, Materials science

## Abstract

Concrete manufacturing makes a major contribution to the world economy, especially the construction industry, but it has a high level of CO_2_ emissions, which harm the environment. To address this environmental issue, this paper investigates the possibility of utilizing a natural mineral that consists of iron, silica, and magnesium, and that is referred to as calcium carbonate, to partially substitute cement. This has been neglected material in past studies, even though it can be used to boost the process of hydration, chemical stability, and even be low in solubility in water. The study is exploring the characteristics of calcium carbonate (CaCO_3_)-modified concrete, such as its physical, rheological, and microstructural properties. CaCO_3_ and ground granulated blast furnace slag were taken as a replacement of 5%, 10%, 15% and 20% of the cement by weight, with a constant water to cement ratio being 0.45, and 50% replacement of cement by ground granulated blast furnace slag (GGBS). The plots of Nyquist and Bode that the microstructural analysis of the changes in the concrete matrix. Non-destructive testing was used to test the performance of the concrete in the long term, quality, homogeneity, and the hardness of the surface. The findings revealed that CaCO_3_-modified concrete was better than traditional concrete, especially in its resistance to carbonation. It gave the best result with 15% cement replacement, where compressive strength was 71.1 MPa, split tensile strength was 4.72 MPa, and flexural strength was 5.89 MPa after 90 days. The results indicate that CaCO_3_ can be an effective alternative to cement that can be used in the production of concrete.

## Introduction

Concrete is the most commonly used construction material in the entire world, which is made of cement, aggregate, water and other chemical admixtures. Its physical, mechanical and durability are the result of intricate interactions of its constituents and mix parameters, such as cement content, water-to-cement (W/C) ratio, water-to-binder (W/B) ratio, curing period, and admixtures dosage, e.g. superplasticizers^1^. The hydration of cement generates calcium silicate hydrate (C–S–H) gels which, among other things, is the source of strength building and densification of the microstructure which, in turn, make the age of curing a crucial parameter of structural performance over time. The differences in these mix parameters are known to have a great impact on the hydration kinetics, porosity development, and the overall load-bearing characteristics of concrete, which further substantiate the significance of microstructure-property relationships in cementitious systems. Researchers have conducted extensive studies to find alternative sustainable building materials that utilize recycled and waste resources because conventional cementitious materials create urgent environmental problems. One promising strategy involves transforming construction and demolition waste into high-quality recycled manufactured sand for mortar applications. The processing of recycled coarse aggregate through crushing to create refined recycled sand produced substantial enhancements to both the mechanical and durability characteristics of sustainable mortar, which proved that high-quality recycled manufactured sand functions as an effective replacement for natural sand in eco-friendly construction materials^2^. The study shows that waste brick materials can be transformed into sustainable geopolymer materials using recycled brick powder, which serves as an environmentally friendly precursor. The research demonstrates that alkali-activated geopolymers made from recycled brick powder achieve improved mechanical and microstructural qualities when researchers use specific silicate modulus and alkali content ratios for their testing^3^. The development of advanced composite materials for sustainable design purposes extends beyond their application in mortar system development. The engineered geopolymer composite system shows that construction waste materials, including recycled concrete powder and paste powder, can be transformed into high-performance ductile materials that need less traditional binder material^4^. The research study investigated the impact of varying percentages and sizes of recycled concrete aggregate materials on the performance of ductile engineered geopolymer composites. The research study confirmed that recycled materials can be utilized in construction materials, which will help develop future building materials^5^. The research community has started to investigate recycling and reuse methods because these methods help decrease construction material environmental impact while simultaneously improving materials performance for building requirements.

The used supplementary cementitious materials (SCM) have drawn the focal point in contemporary concrete technology as these materials have integrated environmental and performance advantages. Depicted mineral admixtures have exhibited activities in pozzolanic reactions with calcium hydroxide (Ca(OH)_2_) to produce more C–S–H, which decreases porosity and increases microstructural density^6^. Their much smaller sizes of particles help them to be less permeable, more durable and resistant in hostile exposure conditions. Sustainability wise, their application also helps to reduce cement usage which is a key factor to take into consideration since cement production directly leads to almost 6–8% of anthropogenic CO_2_ emissions in the world. Therefore, substituting cement with industrial by-products is in line with the global sustainability models and circular economy objectives.

GGBS is among the SCM systems that have received significant contributions to sustainability and performance. Although Ordinary Portland Cement (OPC) manufacture leads to 7–8% of all human-made CO_2_ emissions, GGBS processes also demand much less energy and therefore have a much smaller carbon footprint^7^. The studies of Life Cycle Assessment also confirm that the effects on the environment may be decreased by 50–60 in case of blending GGBS with geopolymer concretes and recycled aggregates. In addition to sustainability, the GGBS will increase the microstructural refinement by creating more C–S–H and Calcium–Alumino–Silicate–Hydrate (C–A–S–H) gels via its latent hydraulic performance. It has been established that nano- and micro-GGBS particles have a significant effect on the formation of strengths, lower sorptivity and resistance to sulfate, chloride and acid attacks with nano-GGBS showing better results owing to its high reactivity^8^. Also reported by techno-economic evaluations is that nano-GGBs concretes are cost-efficient and have better carbon efficiency than the OPC systems. GGBS also carries other advantages to recycled aggregate geopolymer concretes (RAGPC), with its use increasing corrosion resistance, improving surface resistivity, and decreasing carbonation depth-important factors of the long-term viability of reinforced structures^9^. GGBS has a high percentage of Calcium Oxide CaO and semi-amorphous structure, which enables early hydration gel, resistance to acid attack and carbonation, and refinement of the pore. Such properties qualify GGBS to be a critical constituent of sustainable concrete systems in harsh conditions.

Calcium carbonate (CaCO_3_), which is found in various crystalline states (calcite, aragonite and vaterite) has become of great interest in cement science because of its physical filler and chemical stabilizer properties. CaCO_3_ is an active component in the initial hydration and also forms nucleating sites that facilitate the rapid formation of C–S–H and stabilizing the ettringite phases, and hence minimizing porosity^10^. The fact that it is available as limestone or by carbonating industrial by-products provides flexibility, affordability and lower carbon footprint. Nano- CaCO_3_ (NC) with particle sizes of approximately 50 nm and specific surface areas of about 40 m2/g is a good microfiller that reinforces the interfacial transition zone (ITZ) and alleviates weakness in high-strength recycled aggregate concrete (HSRC)^11^. Research also indicates that CaCO_3_ produced in the field of pozzolanic reaction helps in densifying the matrix and strengthening it^12^. Calcium carbide residue (CCR) which has high CaO is also proving to be a promising SCM which is able to boost compressive strength by up to 40% at 5–10% replacement content^13^. Nonetheless, there are still gaps in durability test and microstructural analysis. Simultaneously, the synergistic association of cellulose nanocrystals (CNCs) and nano- CaCO_3_ has been revealed to boost substantially the early-age strength and microstructural refinement by enhancing the NC dispersion and hydration kinetics^14^. Research has shown that the right amount of CaCO_3_ addition (10–15%) enhances compressive strength, but addition of high dosage causes dilution effects and high porosity^15^. On the same note, the addition of CaCO_3_ to porcelain tile waste (PTW) in green self-compacting concrete (SCC) leads to enhanced flowability and mechanical properties, particularly when CaCO_3_ is used at 10–20% and at 30% porcelain tile waste (PTW) content^16^. It has also been used in cemented mine backfill (CMB) systems alongside natural pozzolans, including volcanic ash (VA) and pumicite (PM), where it again tends to increase the strengths of backfilled systems after 28- and 56-day by increasing packing density, and early formation of gel^17^. In addition, CaCO_3_ cement systems, taking advantage of polymorphic transformations and especially vaterite to aragonite transition, show up to 3.5 times high compressive strength associated with needle-like morphologies being interlocked^18^. Simultaneously, mixing GGBS with FA in geopolymer concretes leads to the high compressive and tensile strength with optimum replacement rates of GGBS and FA up to 25 and 20, respectively^19^. Extensive prospects affirm that GGBS enhances the sulfate resistance, chloride penetration resistance, and long durability when applied at the 30–50% substitution rates^20^.

Considering the context of the above, this study presents an environmental-friendly strategy whereby 50% GGBS is partially replaced by an alternative replacement of 50% GGBS coupled with the addition of CaCO_3_ as secondary replacement of GGBS up to 40% though at the same time, natural river sand is replaced by manufactured sand (M-sand). The purpose of this integrated strategy is to minimize carbon emissions and use natural resources without affecting the performance. CaCO_3_ plays roles in enhancing hydration, stabilizing aluminate phases and microstructural modification with the help of its nucleation and filler properties whereas GGBS offers long term pozzolanic reactivity and microstructural densification. Detailed microstructural characterization, electrical impedance spectroscopy (Nyquist and Bode plots), and rheological and non-destructive tests revealed 15% CaCO_3_ to be the best replacement level, which produced a high compressive, tensile and flexural strength with a lower carbonation depth. The incorporation of this holistic material design linked place the study at a considerable level of advancement in the creation of high-performance and sustainable concrete to be utilized in the modern infrastructure works.

## Materials and methods

### Materials

The Ordinary Portland Cement (OPC) 53-grade was selected as the main binder because it is widely accepted in the realm of the structural use and has the capacity to reach a characteristic compressive strength of 53 Mpa at the age of 28 days according to IS 269: 2015^21^. It had a specific gravity of 3.15 according to the IS 2720 (Part 3)^22^, which was good enough to present reliability and consistency in the test of modified cementitious systems. Manufactured sand (M-sand) which was manufactured because of the controlled crushing of hard rock aggregates like basalt was used as a total replacement of natural river sand in accordance with IS 383:1970 23. The M-sand met the criteria of Grade II fine aggregates, and the fineness of the sand was 2.76 with specific gravity of 2.7, and the coarse aggregate that was produced (20 mm size) has specific gravity of 2.8 with 0.4 per cent absorption of water according to IS 2386 (Part III):1963. Angularity and well-graded quality of M-sand improve the packing, interfacial bonding, and strength of the particle and is thus especially prone to high-performance concretes^23^. In order to have sufficient workability and dispersion of cementitious particles in mixes with lower water content, polycarboxylate ether (PCE)-based superplasticizer (Novamix 4000, Chemtech Concrete India Pvt. Ltd.) was used at the level of 0.5% of cement. The combination of OPC, well-graded M-sand, durable coarse aggregates, and an advanced PCE admixture offers an excellent starting point in exploring the behavior of CaCO_3_-modified cementitious systems based on their carboxyl (COO–) and sulfonate (SO_3_)-based functional groups which demonstrate excellent steric hindrance and electrostatic repulsion mechanisms resulting in efficient dispersion to reduce viscosity, enhance flowability, and control cracking and determination of long-term durability.

#### Granulated Blast Furnace Slag

GGBS (Ground Granulated Blast Furnace Slag) is a by-product of the manufacturing of iron and steel and serves as a very effective supplementary cementitious material (SCM) to sustainable concrete. In general, GGBS can be used as a substitute to 20–70% of cement, which has high environmental and performance advantages^24^. GGBS, as indicated in Table 1, includes more SiO_2_ (30–40%), Al_2_O_3_ (10–15%), and MgO (5–10%), than OPC, and thus, improved pozzolanic and latent hydraulic reactions, which lead to further C–S–H development and better microstructural densification. It has a smaller particle size, which enhances easy packing, low connection between pores, and high strength development during use. GGBS concrete also has better strength especially resistance to sulfate attack, chloride penetration, and alkali-silica reaction, and therefore it can be used in marine and chemically aggressive environments. Moreover, it has low heat of hydration that reduces thermal cracking when used in mass concrete. Even though there could be a delay in early-age strength which is caused by a lower reactivity, this can be efficiently countered by employing a correct curing regime. With its stability in chemical structure, lower carbon footprint and extended durability, GGBS will continue to be a major constituent in the construction of low-carbon and eco-efficient concrete systems.

#### Calcium carbonate (CaCO_3_)

Calcium carbonate (CaCO_3_) is a naturally occurring inorganic compound composed of calcium and carbonate ions, widely distributed in geological formations such as limestone, marble, and marine shells. Its chemical composition, including high CaO content (52–56%) and minimal silica and alumina impurities, as presented in Table 1, contributes to its stability and compatibility with cementitious systems. In India, CaCO_3_ is abundantly available and extensively utilized across construction and cement-based industries, owing to its physical durability, chemical inertness, and low environmental footprint^25^. In the present study, CaCO_3_ powder was incorporated into concrete to evaluate its influence on mechanical performance and sustainability. The specific gravity of CaCO_3_ was determined as 2.65 using the pycnometer method in accordance with IS 2720 (Part-3). Its fine particle size enables CaCO_3_ to act as an efficient microfiller, enhancing concrete microstructure by refining pore networks, improving particle packing, and reducing void content. This filler action leads to smoother surface finishes, improved cohesiveness, and better pumpability^26^. Additionally, CaCO_3_ enhances workability by increasing slump and accelerating early hydration through nucleation effects, while its interaction with aluminate phases contributes to carboaluminate formation, stabilizing ettringite and improving long-term durability. By partially replacing cement, CaCO_3_ reduces clinker demand, thereby lowering embodied carbon and supporting sustainable construction practices without compromising early-age or long-term strength development.

**Table 1:** Chemical composition of cement, GGBS, and calcium carbonate.

Element	Chemical compounds in %		
	Cement	GGBS	Calcium carbonate
CaO	60–67	30–45	52–56
SiO_2_	17–25	30–40	0.1–2
Al_2_O_3_	3–8	10–15	0.5–1
Fe_2_O_3_	0.5–6	0.5–2	0.05–1.5
MgO	0.1–4	5–10	0.2–2
Alkalis	0.2–1.3	< 1	0.05–0.5
SO_3_	1–3	1–3	0.01–0.5
TiO_2_	0.1–0.4	< 1	0.01–0.2
LOI	< 3	< 3	42–44

#### Particle size distribution analysis (PSD)

**Fig. 1:**
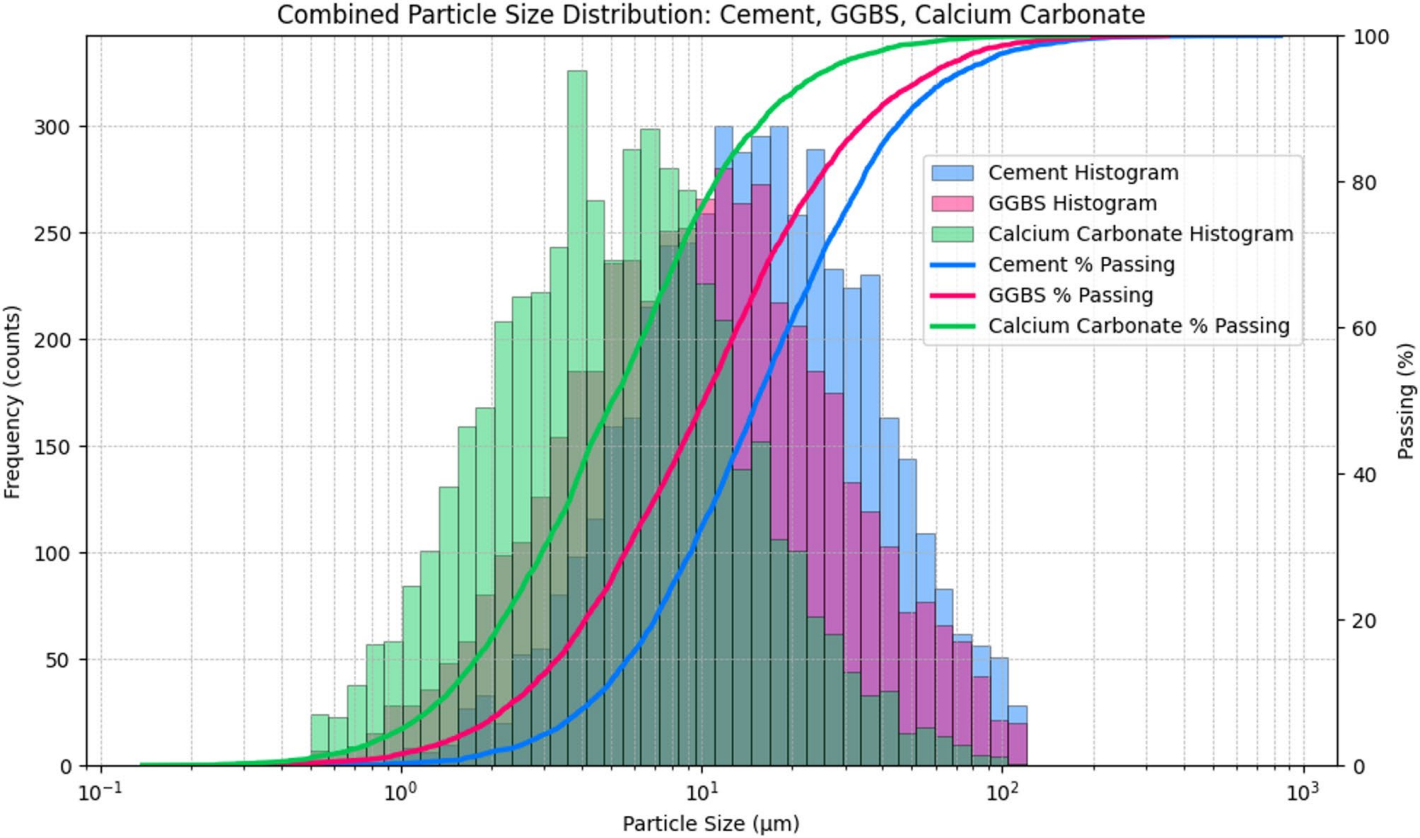
Combined particle size distribution analysis.

The significant differences in fineness and gradation shown by the particle size distribution (PSD) analysis of cement, GGBS, and calcium carbonate powder is a clear indication that the gradation and fineness differences significantly affect packing density and composite binder behavior (Fig. 1.). PSD curves developed using the histogram indicates that the cement has the coarsest distribution with a wide distribution of particles ranging between 5 and 80 μm, the GGBS has moderately finer distribution with level ranging between 3 and 40 μm, and calcium carbonate power had the finest fraction with majority of the particles between 1 and 20 μm. These differences are further substantiated by the empirical cumulative passing curves where CaCO_3_ is 90% passing at a size of about 12 μm, GGBS is 30 μm and cement is 35 μm, which confirms these two materials, CaCO_3_ and GGBS, are microfillers and cement is intermediate. As there has been no mathematical smoothing done, the PSD curves represent the real measured distribution of particles without any approximations that give a realistic picture of material gradation as used in concrete batching. According to these features of PSD, it is scientifically proven that the partial substitution of cement with GGBS and CaCO_3_ is possible. The smaller GGBS particles occupy spaces between the larger cement particles, increase the density of packing, decrease capillary porosity, increase strength after later ages by secondary C–S–H formation, and decrease heat of hydration^27^. The ultra-fine CaCO_3_ particles, in the meantime, serve as good micro fillers and nucleation sites, which hastens early hydration, enhances cohesiveness as well as decreased water demand^28^. The three materials make a sensible multi-modal PSD which maximizes binder system particle packing, leading to denser microstructure, lower permeability, and enhanced mechanical performance. The PSD analysis therefore gives a solid reason as to why ternary blending of cement and GGBS and CaCO_3_ is a sustainable, effective and performance enhancing method of cementitious composites in the modern world.

#### Preparation of calcium carbonate paste

The process of preparing calcium carbonate (CaCO_3_) paste is based on mixing the raw CaCO_3_ powder with water to create an aqueous mixture of two particles through the physical interactions between the water and the particle. In typical circumstances, CaCO_3_ is chemically inert and has a very low solubility in water thus there are no essential hydration and cementitious reactions as the paste is being prepared. The fresh paste can be observed to be cohesive due to forces of surface tension, capillary water-films and mechanical interlocking between the fine particles. Where trace impurities (e.g. calcium oxide (CaO), magnesium oxide (MgO) are present—as indicated by the common chemical compositions (Table 1)—a slight setting tendency can be obtained after standing 24 h because these reactive oxides are hydrated. Causal minor stiffening can also be due to moisture loss, compaction of particles or weak carbonation by atmospheric CO_2_, but these effects are minimal in pure CaCO_3_ systems. The fine surface cracks are such that they are common in the paste in the first 24 h. These cracks are explained by the process of different drying shrinkage, in which water moves upwards and evaporates faster at the open surface to accumulate tensile stresses which surpass the low adhesive strength of the CaCO_3_ matrix^17^. Other factors could be internal micro-voids, nonuniform water distribution, and localized volume changes that may be applied due to minor chemical reactions related to impurities. CaCO_3_ is non-pozzolanic and does not contain any reactive silica or alumina which means that alone it cannot form any binding products to oppose these stresses and its structural stability and cracking behavior is thus limited. These deficiencies can be effectively caused by the inclusion of cement to CaCO_3_ paste to produce a reactive and binding matrix that can form calcium silicate hydrate (C–S–H) gel in the process of hydration^11^. The cement filling Micro-voids is occupied by cement hydrates that greatly enhance the packing density reducing drying shrinkage and enhancing the overall structural integrity. Even though CaCO_3_ is not a species that is involved in pozzolanic reactions, its ultrafine particles are a highly efficient filler and nucleation media that facilitate the deposition of hydration products and early C–S–H formation via a heterogeneous nucleation process. This synergistic effect of cement hydration and CaCO_3_ filler effects lead to the attainment of lower crack formation, high volumetric stability, and quantifiable advantages in the strength of concrete over extended curing duration. Therefore, the adjustment of CaCO_3_ paste using cement does not only counter the inert behavior of CaCO_3_, but also converts the mixture into a sound cementitious skeleton, which exhibits better microstructural stability and mechanical serviceability that will be applicable in sustainable buildings construction.

#### Calcium carbonate modified cement: standard consistency and setting time behavior

**Fig. 2:**
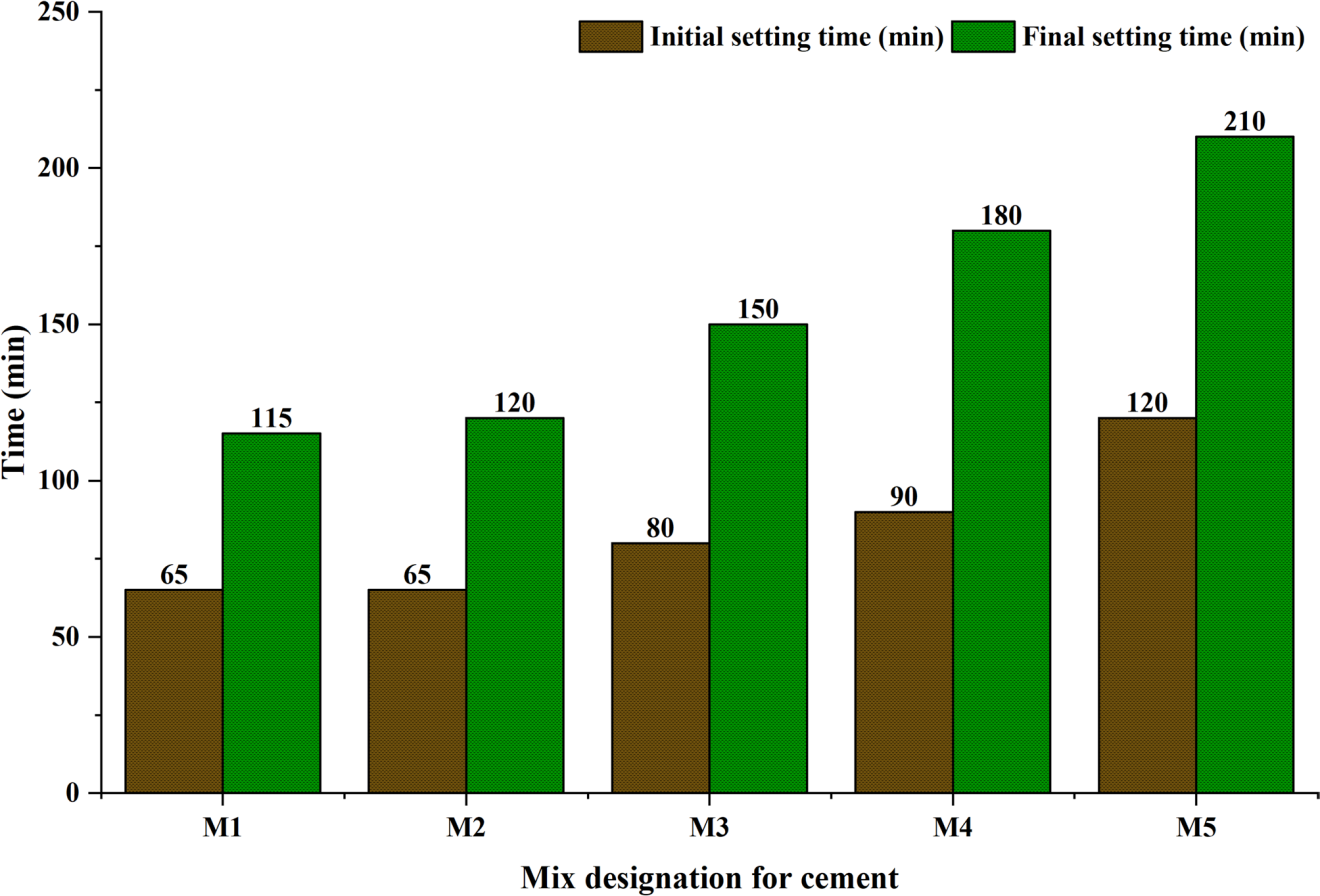
Initial and final setting times of calcium carbonate modified cement.

The influence of calcium carbonate (CaCO_3_) on the consistency and setting characteristics of cementitious systems was examined through a series of tests conducted in accordance with IS: 4031 (Part 4) 1988 and IS: 4031 (Part 5) 1988. Standard consistency was determined by preparing pastes using 400 g of cementitious materials and varying water contents until the Vicat plunger achieved a penetration depth of 5–7 mm, ensuring adequate workability and uniformity. As shown in Table 2, the incorporation of CaCO_3_, owing to its finer particle size and chemical composition, altered the water demand of the mixes, requiring incremental adjustments in the percentage of water to achieve standard consistency. Setting time analysis further revealed that the addition of CaCO_3_ progressively increased both the initial setting time (IST) and final setting time (FST) up to an optimal dosage range^29^. Specifically, CaCO_3_ additions of 5–20% produced IST increases of 8–44% and FST increases of 8.84–32.09%, primarily due to clinker dilution and reduced availability of reactive C_3_S and C_3_A phases, which collectively retard hydration and delay C–S–H gel formation. At higher dosages (> 20%), the setting times began to decline, a behavior attributed to microstructural densification, void filling, and enhanced nucleation, which provided greater resistance to needle penetration. In this study, CaCO_3_-modified OPC paste exhibited IST and FST increases of up to 25% and 27.5%, respectively, demonstrating a clear dosage-dependent effect on cement setting behavior. The complete setting time response for all mixes (M1–M5), prepared as per the proportions listed in Table 2, is shown graphically in Fig. 2, confirming the dual influence of dilution-induced retardation at lower dosages and particle-packing-induced acceleration at higher dosages.

**Table 2:** Mix design of CaCO_3_ modified cement.

Mix design	Cement [kg/m^3^]	GGBS [kg/m^3^]	CaCO_3_ [kg/m^3^]	*P* (% of water content from standard consistency)	Water content (0.85P of cementitious material) [kg/m^3^]
M1	190	190	0	32	108.8
M2	190	171	19	32	108.8
M3	190	152	38	33	112.2
M4	190	133	57	33	112.2
M5	190	114	76	34	115.6

### Methods

**Fig. 3:**
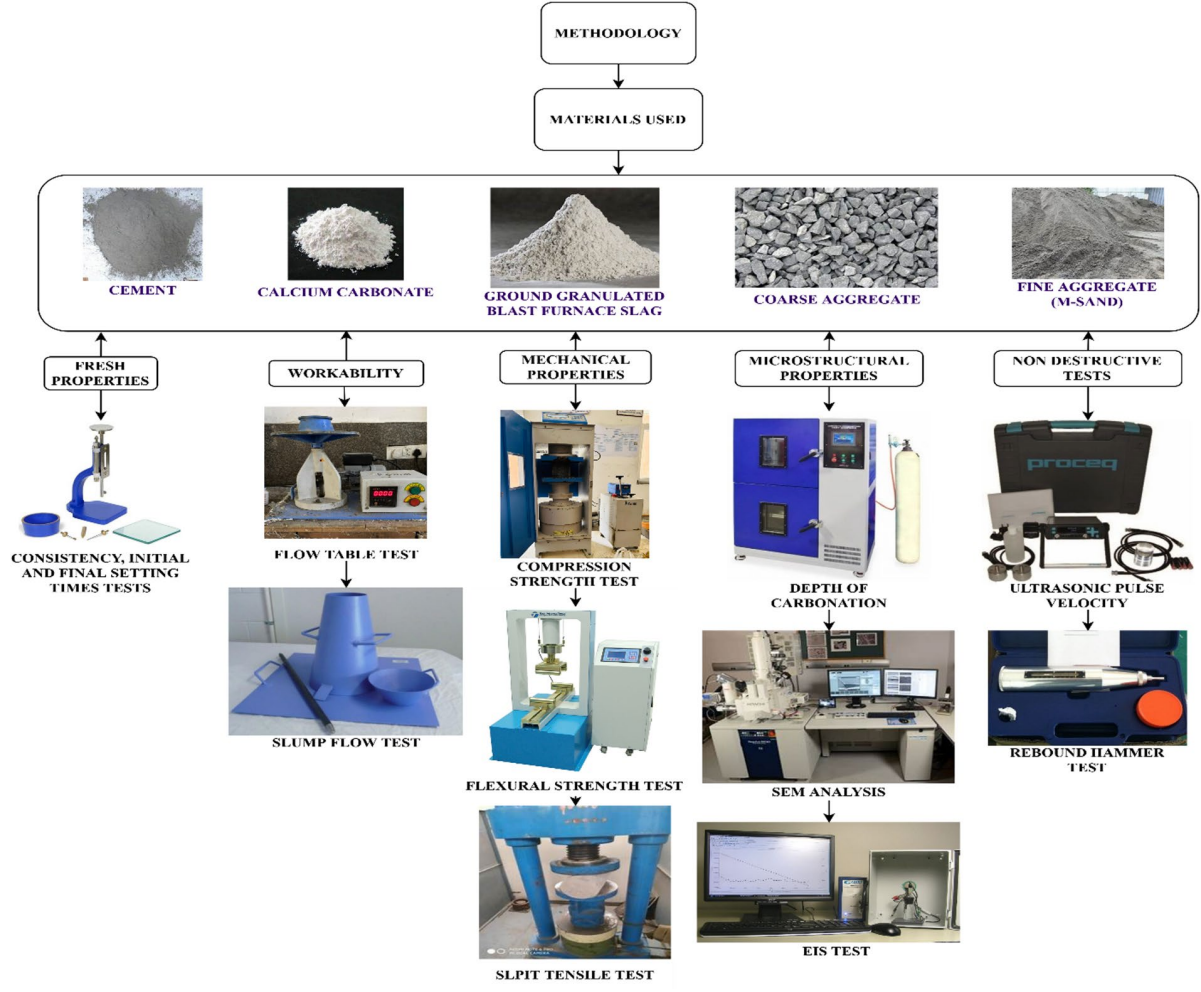
Methodology of the present study.

The experimental program was based on a systematic working process, which is shown in Fig. 3, to determine the effects of calcium carbonate (CaCO_3_) and GGBS on fresh, mechanical, and microstructural performance of concrete. First, the physical traits of the constituent materials such as CaCO_3_, fine aggregate (M-sand), and coarse aggregate were described in line with the respective IS standards taking specific attention to the criteria of particle gradation, specific gravity, water absorption, and material conformity. Concrete mixtures of CaCO_3_ and GGBS were then done with concrete mixtures with replacement levels indicated, and the fresh properties of the mixtures were evaluated using standard consistency measurement, initial and final setting time test, flow table test, and slump flow behavior. Hardened concrete samples were then subjected to a complete package of mechanical tests i.e., compressive strength, flexural strength and split tensile strength to identify how CaCO_3_ modified mixes would respond to a load and carry the weight. Also, depth of carbonation measures, ultrasonic pulse velocity (UPV), rebound hammer test and electrical impedance spectroscopy were used to study durability related and microstructural properties. This combined methodological approach made the rigorous analysis of the performance development of the modified concrete, its fresh condition to the hardened and microstructurally developed state possible.

#### Microstructural and electrochemical characterization

The microstructural characteristics of the CaCO_3_ modified cementitious systems were examined using Scanning Electron Microscopy (SEM) coupled with Energy Dispersive X-ray Spectroscopy (EDS), which together provide high-resolution imaging and precise elemental analysis essential for interpreting phase morphology and hydration behavior. SEM techniques, including secondary electron (SE) and backscattered electron (BSE) imaging were employed to obtain two-dimensional digital micrographs that reveal surface texture, pore topology, and the distribution of hydration products. BSE imaging further enhanced compositional contrast, enabling visualization of dense and porous regions, while EDS mapping facilitated elemental quantification and identification of hydrate phases, particularly where differentiating between AFm variants or calcium-rich phases is required^30^. Data post-processing involved noise reduction, segmentation (e.g., SLIC algorithm), and atomic ratio evaluation to construct composite microstructural maps depicting the spatial arrangement of hydration products, pores, and CaCO₃ particles. SEM–EDS analysis using a ZEISS microscope used to confirm that concrete and mortar containing CaCO_3_ exhibited a denser microstructure with improved void filling compared to the control mix. Increased CaCO_3_ content resulted in more compact morphologies due to the filler effect and enhanced nucleation, contributing to reduced porosity and improved structural integrity. These findings substantiate the role of CaCO_3_ as an effective microstructural modifier that enhances both durability and mechanical performance.

To further investigate the internal structure and ion transport behavior of the modified concrete, Electrochemical Impedance Spectroscopy (EIS) was conducted as a non-destructive diagnostic technique. EIS analyzes frequency-dependent electrical impedance responses to quantify microstructural evolution, pore connectivity, and electrochemical durability indicators^26^. Concrete specimens fitted with embedded copper-wire electrodes were connected to an electrochemical workstation, where a low-amplitude alternating current (AC) signal was applied across parallel electrodes over a broad frequency range. The resulting impedance spectra including Nyquist and Bode plots were used to evaluate resistance, capacitance, and charge-transfer characteristics associated with hydrated phases and ionic pathways. EIS provided insight into changes in electrical resistivity, moisture transport, and microstructural refinement associated with CaCO_3_ incorporation, while also enabling the assessment of carbonation progression and durability. The technique’s sensitivity to pore structure and ionic composition makes it a powerful tool for understanding the long-term performance of CaCO_3_-modified cementitious materials, especially in systems designed for enhanced sustainability and reduced carbon footprint. Together, the combined SEM–EDS and EIS analyses offer a comprehensive understanding of how CaCO_3_ influences microstructure, densification, and electrochemical stability in cementitious matrices.

## Results and discussion

### Calcium carbonate modified cement mortar

#### Workability of by flow table test

**Fig. 4:**
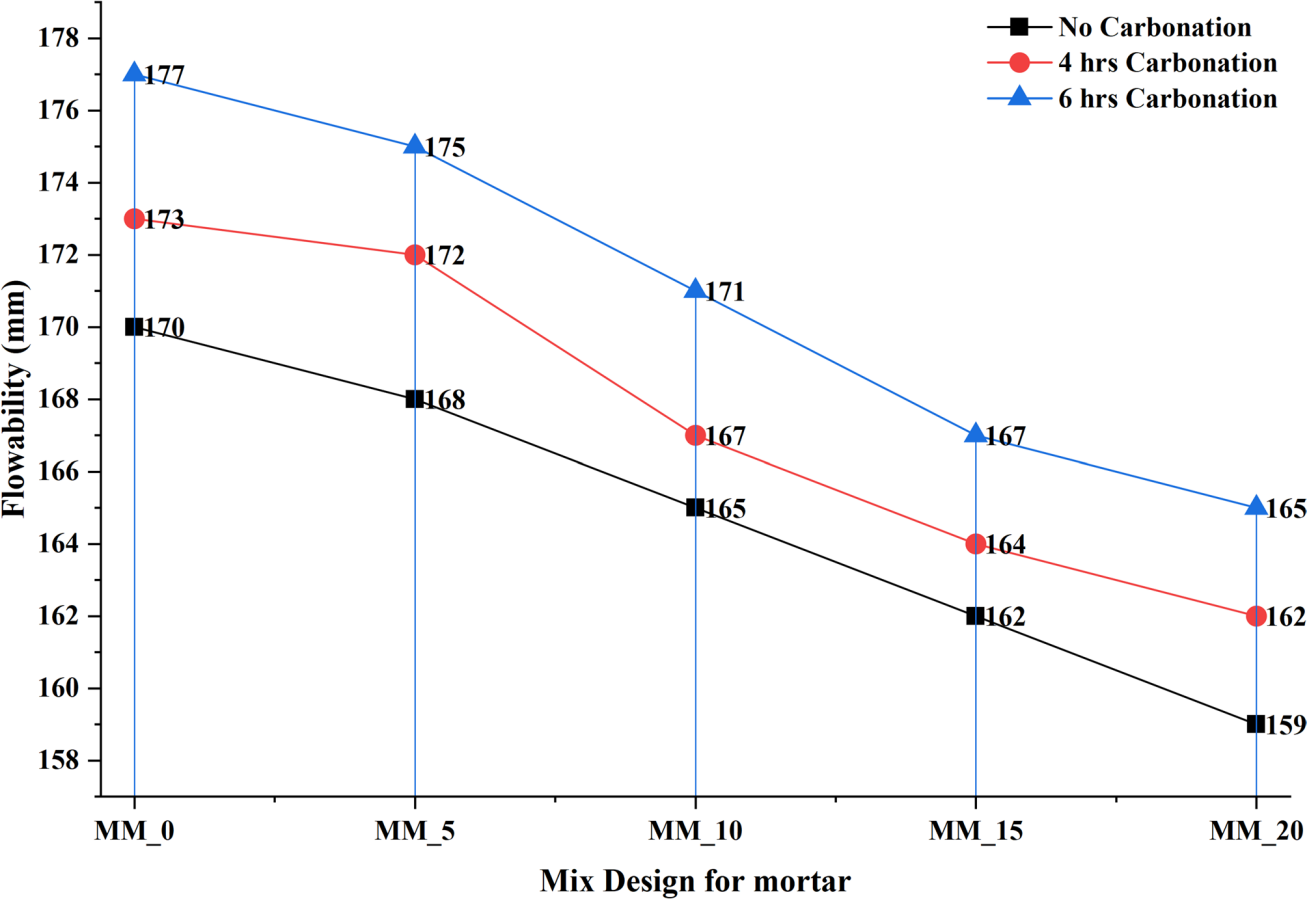
Flowability of calcium carbonate modified cement mortar.

The spread of a 1:3 mixture of cement sand made according to the requirement of the IS 1199:1959 31 was used to evaluate the workability behavior of CaCO_3_ modified cement mortar using the flow table test. Table 3 shows the mortar compositions of various replacement levels of CaCO3 (MM_0 to MM_20), whereas the value of flow, measured with varying carbonation times (0 h, 4 h, and 6 h), is indicated in Fig. 4. As shown in the results, the flowability decreased steadily with the addition of CaCO_3_ content under all carbonation conditions. In the case of the non-carbonated mixes, flow reduced with increasing CaCO_3_ level because MM_0 had a flow of 170 mm, and MM-20 had a flow of 159 mm. This is mainly because the CaCO_3_ fine particles have high specific surface area and water requirement, and therefore, reduce the free water available to lubrication^19^. The same declining pattern can be seen with under 4-hour carbonation with flow values of 173 mm (MM_0) and 162 mm (MM_20). Subtly, carbonation is found to enhance the workability of the mixes that are not carbonated because of the calcium bicarbonate films formed which serve as temporary lubricating agents. The greatest flow values were recorded at less than 6-hour carbonation, falling as 177 mm (MM_0) to 165 mm (MM_20) meaning that long period carbonation increases wetting and particle dispersion leading to enhanced mobility. Nonetheless, flow decreases steadily with the addition of increasing CaCO_3_ content with or without carbonation, which proves that the filler effect prevails over the lubrication effect of carbonation^31^. All in all, the findings show that although carbonation enhances the workability of all mixes, increased rates of CaCO_3_ replacement inevitably reduce the flowability because of the finer nature and water absorption. Such a tendency can be explained by the fact that fine powders require more mixing water to be consumed. Although this decrease was evident, the flow values are still within acceptable range to show that when water adjustment is done according to Table 3, CaCO_3_ can be incorporated adequately without affecting mortar workability. The given behavior explains why one has to optimize the content of water or the dosage of superplasticizer when increasing the proportions of CaCO3 to preserve the desired fresh properties.

**Table 3:** Mix design of CaCO_3_ modified mortar.

Mix design	Cement (kg/m^3^)	GGBS (kg/m^3^)	CaCO_3_ (kg/m^3^)	Manufacture sand (kg/m^3^)	Water content (kg/m^3^)
MM_0	190	190	0	1140	103.36
MM_5	190	171	19	1140	103.36
MM_10	190	152	38	1140	106.59
MM_15	190	133	57	1140	106.59
MM_20	190	114	76	1140	109.82

#### Compressive strength of calcium carbonate modified cement mortar

**Fig. 5:**
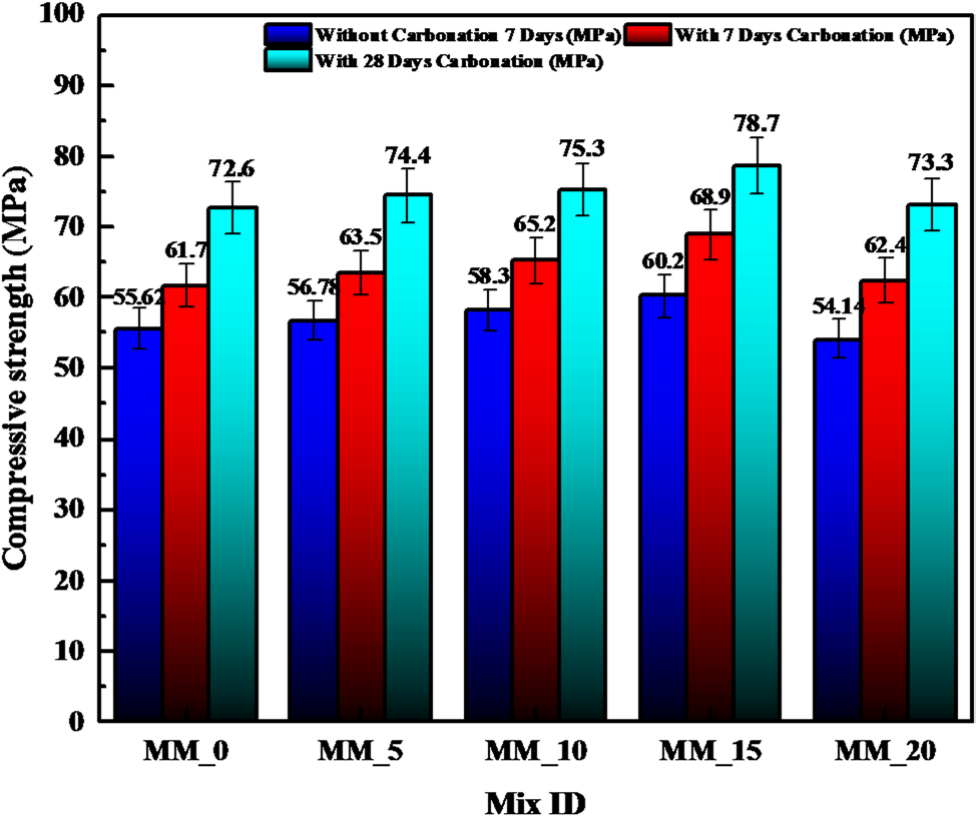
Compressive strength CaCO_3_ modified cement mortar.

**Fig. 6:**
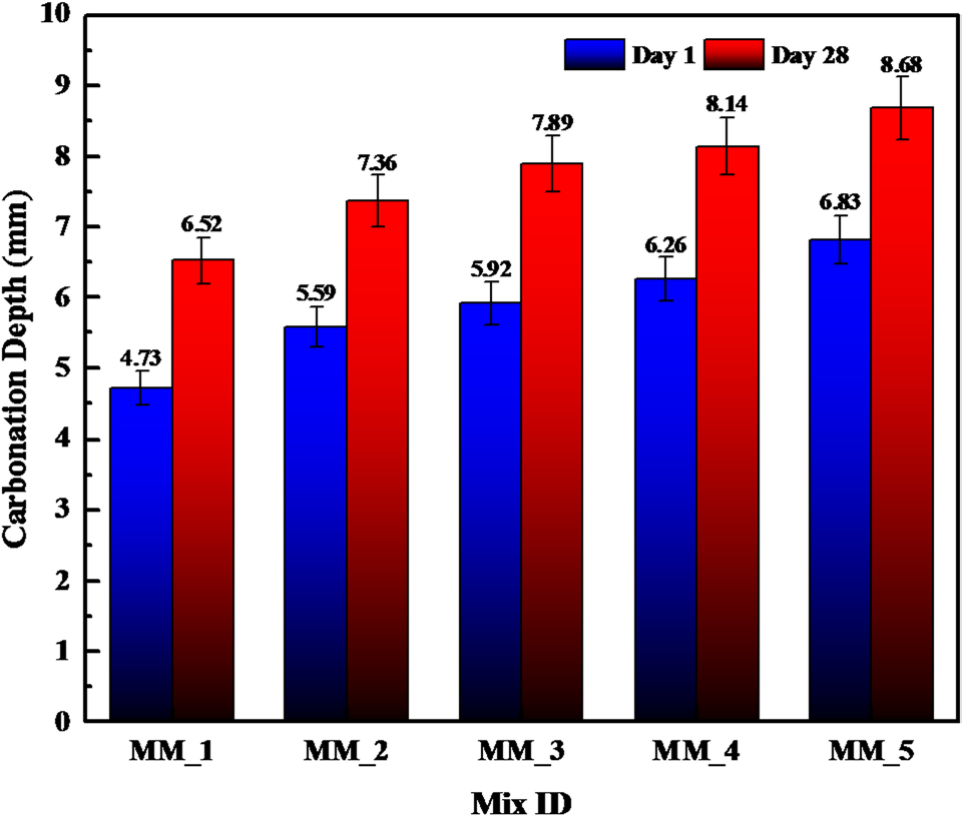
Depth of carbonation for 1 day and 28 days of carbonation of specimens.

Figure 5 shows the compressive strength behavior of CaCO_3_ -modified cement mortar in various carbonation regimes including no carbonation, 7 days carbonation and 28 days carbonation. Strength is also steadily increased with the addition of CaCO_3_ and the increase is even more marked when accelerated carbonation (10% CO_2_, 60% RH, 2 bar, 27 +- 3 °C) is used. In such conditions, CaCO_3_ is shown to exhibit limited inherent chemical reactivity, though surface recrystallization to form secondary layers of CaCO_3_ is observed and as such helps to increase the strength of the material by 10–15% in 28 days of time again it is seen that earlier carbonation-based studies had found the same^15^. In all mix designations (MM_0-MM_20), non-carbonated samples exhibit the moderate strength gains owing to the familiar filler effect of CaCO_3_. Its fine grains can effectively fill micro-pores, refine pore structure and facilitate early hydration through nucleation sites in CSH formation, to give denser microstructures^32^. This explains the small yet steady increase in the red bars of Fig. 6. But as the mortar is exposed to CO_2_ the strength gain is much greater. In the case of carbonate, the reactant is CO_2_ which reacts not only with calcium hydroxide, but also with calcium bearing phases formed as a result of the CaCO_3_ alteration, and thus forms stable carbonate precipitates like calcite and aragonite. Such precipitates occupy the remaining pore spaces, decrease the capillary porosity, and strengthen the microstructure, which explains the massive increase observed in both the 7-day and 28-day carbonation outcomes. The maximum values of compressive strength were produced in MM_15 (78.7 MPa, 28 days carbonation) which means that the ideal balance between the content of CaCO_3_, the arrangement of particles and formation of carbonation products was achieved. At this level (MM_20), the marginal decrease in strength can be explained by the excessive concentration of CaCO_3_ which leads to dilution of reactive cement phases and the reduction of hydration potential despite the advantages of carbonation^15^. The presence of fine CaCO_3_ particles leads to changes in the pore network because they create more complex paths, which result in slower CO_2_ movement and decreased carbonation depth throughout the years. The carbonation coefficient (K) shows an increase with elevated water-to-binder ratios because higher ratios enable better pore connectivity, yet the addition of micro- and nano-size CaCO_3_ particles leads to enhanced material density through matrix densification, while it decreases permeability. The dual functionality of CaCO_3_ as an additive allows it to improve early strength through its filler and nucleation capabilities while it strengthens later stages through carbonation-based pore enhancement. The high gains in strength in accelerated carbonation in Fig. 5 indicate that CaCO_3_ is effective in conjugating CO_2_ sequestration and, at the same time, improving the mechanical strength of cement mortars, and its applicability is therefore recommended in sustainable and durable cementitious systems.

#### Depth of carbonation of calcium carbonate modified cement mortar

CaCO_3_-modified cement mortar was tested in terms of carbonation through the casting of 70.6 mm cubes of mortar, and the experiment was carried out on accelerated carbonation over 1 day in a controlled chamber. Sixty specimens were used so that they could be statistically reliable. Upon exposure, the cubes were broken and sprayed using a phenolphthalein indicator to identify carbonated and non-carbonated areas. The carbonated zones had no color since the alkalinity was low (pH less than 9.5), though, the uncarbonated regions had a specific purple-red color, which was an indication of the presence of alkaline phases like Ca(OH)_2_. The depth of the carbonation at various locations of the fractured surface was then measured with Vernier calipers, and the mean depth of the carbonation in each mix was then displayed in Fig. 6. The findings show that carbonation depth is increasing with the presence of more CaCO_3_ where Day 1 values are 4.73 mm (MM_1), and the values 6.52 mm to 8.68 mm appeared after 28 days. This is noted to be due to the observed dilution effect of the partial replacement of cement by CaCO_3_ which lowers the quantity of reactive calcium hydroxide to buffer the ingress of CO_2_^31^. Simultaneously, smaller CaCO_3_ particles augment pore discontinuity, but can also augment capillary connectivity, enabling accelerated CO_2_ infiltration in elevated replacement mixes. Also, the lowering of the C–S–H gel formation as a result of lowering clinker content lowers the alkalinity of the matrix and increases the speed of the carbonation front. Nevertheless, the carbonation depths are not excessive to modified mortar, which suggests that CaCO_3_ does not negatively affect the durability in case it is applied in the required amounts. This tendency of Fig. 8 agrees with literature on carbonation in systems with non-pozzolanic fillers, where alkalinity and microstructural density reductions are found to moderate carbonation tendencies. These data show that the optimal dosage of CaCO_3_ should be used to balance mechanical performance, micro structural refinement and long-term durability.

.

#### Scanning electron microscopy (SEM) of mortar

**Fig. 7:**
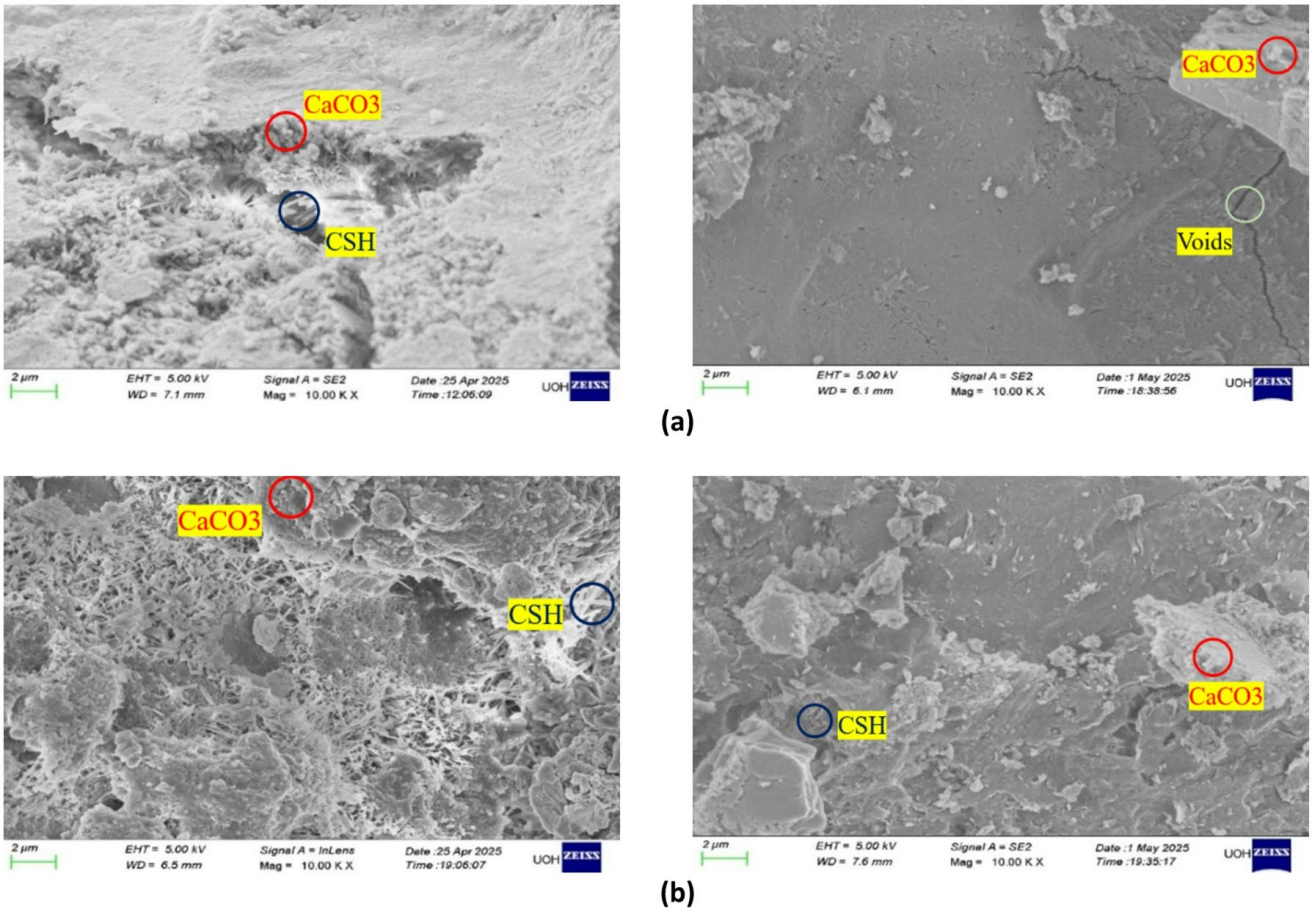
Scanning electron microscopy of mortar (**A**) Optimum without carbonation and (**B**) Optimum with carbonation.

Microstructural development of CaCO_3_-modified cement mortar was observed in a Field Emission Scanning Electron Microscope (FESEM) at magnification of 10,000× so that hydration products and morphology of pores could be visualized clearly. The non-carbonated specimens have a highly porous structure as shown in Fig. 7a which is in the form of loosely packed calcium silicate hydrate (C–S–H) gels, interconnected gaps and microcracks. These characteristics suggest that it is more permeable and less compact, both of which tend to suggest a low filler action and incomplete hydration. The presence of discrete particles of CaCO_3_ incorporated in the matrix also indicates that they are inert fillers without carbonation and do not have substantial bonding to adjoining hydrates^33^. Conversely, Fig. 7b shows that the micrographs of carbonated mortar using the SEM technique show significant densification and structural refinement. The carbonation of calcium hydroxide in controlled CO_2_ environment enhances the creation of secondary CaCO_3_ which is well distributed carbonate precipitates that can effectively fill the micro-pores and micro-cracks. These precipitates which appear as the dense crystalline aggregates enhance the interlocking of C–S–H phases and homogeneity of the matrix^34^. The decrease in pore continuity and creation of highly crowded C–S–H network illustrates the evident increase of microstructural integrity following carbonation. The densification that is observed is consistent with the chemical process according to which CO_2_ reacts with accessible calcium phases to form stable compounds of carbonate and this causes less porosity and increases durability. In sum, the fact that carbonation significantly improves the quality of the microstructure of CaCO_3_-modified mortar by accelerating the filling of the voids, enhancing the packing density, as well as improving the strength of the interfacial transition zone proves that carbonation can be used to improve the quality of CaCO_3_-modified mortar. This refined microstructural characteristics directly lead to the increased durability, decreased permeability and increased mechanical performance observed in the carbonated mortar samples which underline the positive synergy between CaCO_3_ modification and the controlled carbonation treatment in cementitious systems.

### Calcium carbonate modified concrete

#### Development of concrete with calcium carbonate

The concrete mixture of CaCO_3_ was planned in accordance with IS 10262: 2019^34^ based on M30 grade concrete at the constant water to binder ratio of 0.45 and the superplasticizer dosage of 0.5% of cementitious material. Control mix (CCM_0) was a 50% OPC and 50% GGBS mix, and modified mixes (CCM_5-CCM_20) gradually substituted GGBS with 5–20% CaCO_3_. The entire proportions of the mix are contained in Table 4. It aimed at lowering the CO_2_ emissions by adding CaCO_3_ without affecting performance. Slump of the mixes was found to be 120 mm, which means good workability and this is because CaCO_3_ has a fine particle size and can enhance the packing density. To conduct performance assessment, each mix was cast and tested at 7, 28, 56 and 90 days and three most consistent samples were used to conduct the final analysis. This provided sound evaluation of the effect of CaCO_3_ on the strength growth and general behavior of the modified concrete.

**Table 4:** Mix design of CaCO_3_ modified concrete.

Mix design	Cement (kg/m^3^)	GGBS (kg/m^3^)	CaCO_3_ (kg/m^3^)	Manufacture sand (kg/m^3^)	Coarse aggregate (kg/m^3^)	Water content (kg/m^3^)
CCM_0	190	190	0	820	1090	171
CCM_5	190	171	19	820	1090	171
CCM_10	190	152	38	820	1090	171
CCM_15	190	133	57	820	1090	171
CCM_20	190	114	76	820	1090	171

#### Workability of concrete by slump flow test

**Fig. 8:**
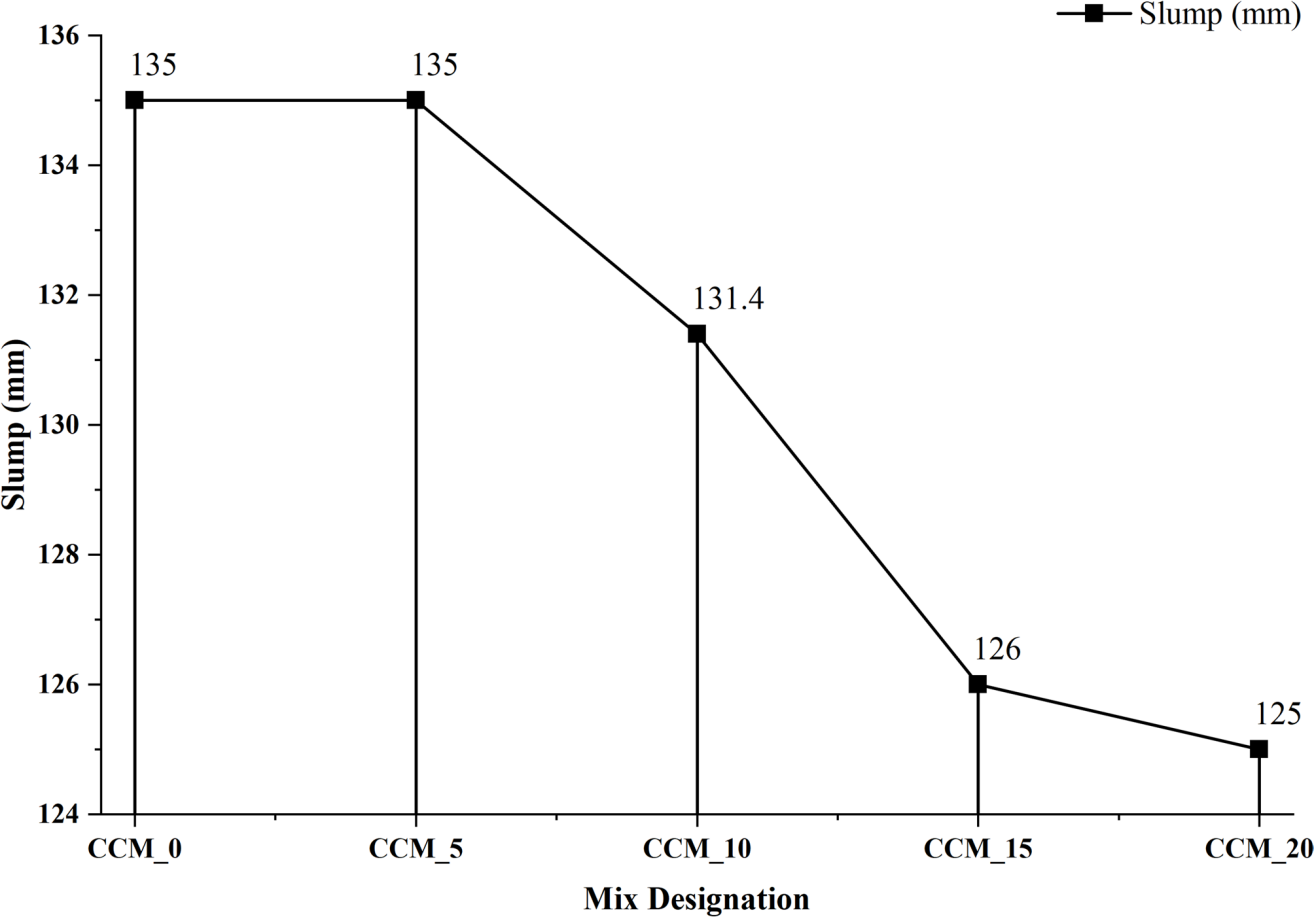
Variation of slump flow of concrete.

Moderator workability of the CaCO_3_-modified concrete was assessed on the basis of the standard slump test as per IS 1199:1959^31^ Fresh concrete was placed in slump cone in three equal layers and compacted with 25 tamping strokes each then the slump cone was raised vertically to enable free deformation of the concrete. The ensuing values of slump of the mixes CCM_0 to CCM_20 are presented in Fig. 8, which clearly shows that the slump decreases with increase in the amount of CaCO_3_. Control mix CCM_0 had a slump of 135 mm which was maintained in CCM_5 because only slight decrease in particle fineness was observed with low dosage of CaCO_3_. Nonetheless, the slump reduced to 131.4 mm in the case of CCM_10 and went further to 126 mm and 125 mm in CCM_15 and CCM_20 respectively. The reason of this decreasing trend is given by the high fineness and high specific surface area of CaCO_3_ particles, which raise water demand, and lower the amount of free water to flow. The higher the CaCO_3_ replacement, the greater absorption of mixing water on the particle surface, which leads to a stiffer mix. Despite the addition of a polycarboxylate-based superplasticizer to ensure the cohesiveness and flowability, high content of CaCO_3_ restrains its dispersive capability because of more adsorption of the admixture on small particles. Throughout, CaCO_3_ enhances particle packing in moderate doses, however, at high doses (15–20%), particle packing would be too thick thus limiting the movement of the paste, and reducing slump.

Although, the percentages of slump decreased, all of the slump values were within the reasonable working range of workable concrete, showing that in proper dosage, CaCO_3_ can be adopted as a sustainable microfiller without affecting fresh characteristics^35^. The findings in Fig. 8 thus confirm the appropriateness of CaCO_3_ as a partial replacement cement as long as suitable adjustments in admixtures are done to maintain desirable workability.

#### Compressive strength test of concrete

**Fig. 9:**
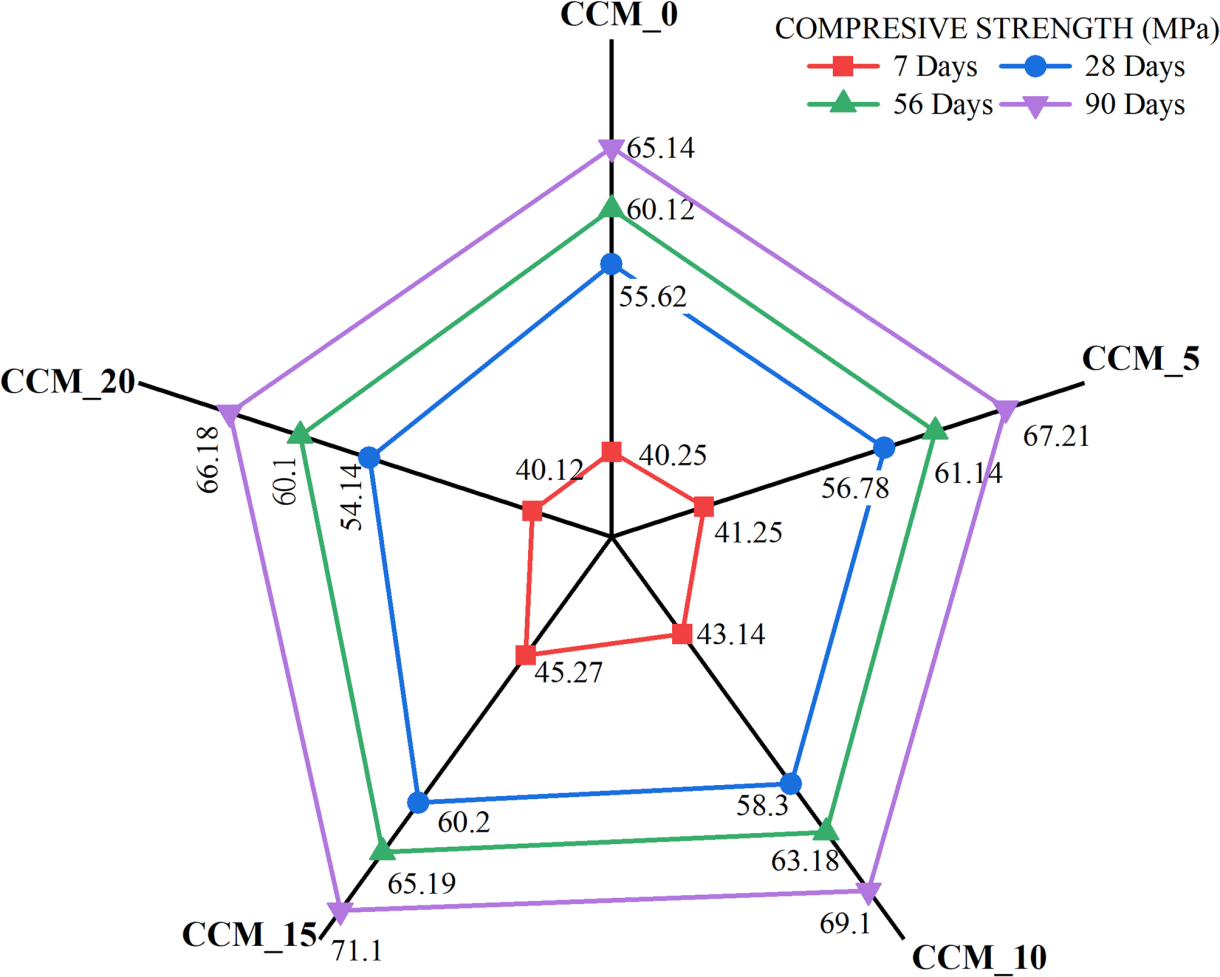
Variation in compressive strength.

**Fig. 10:**
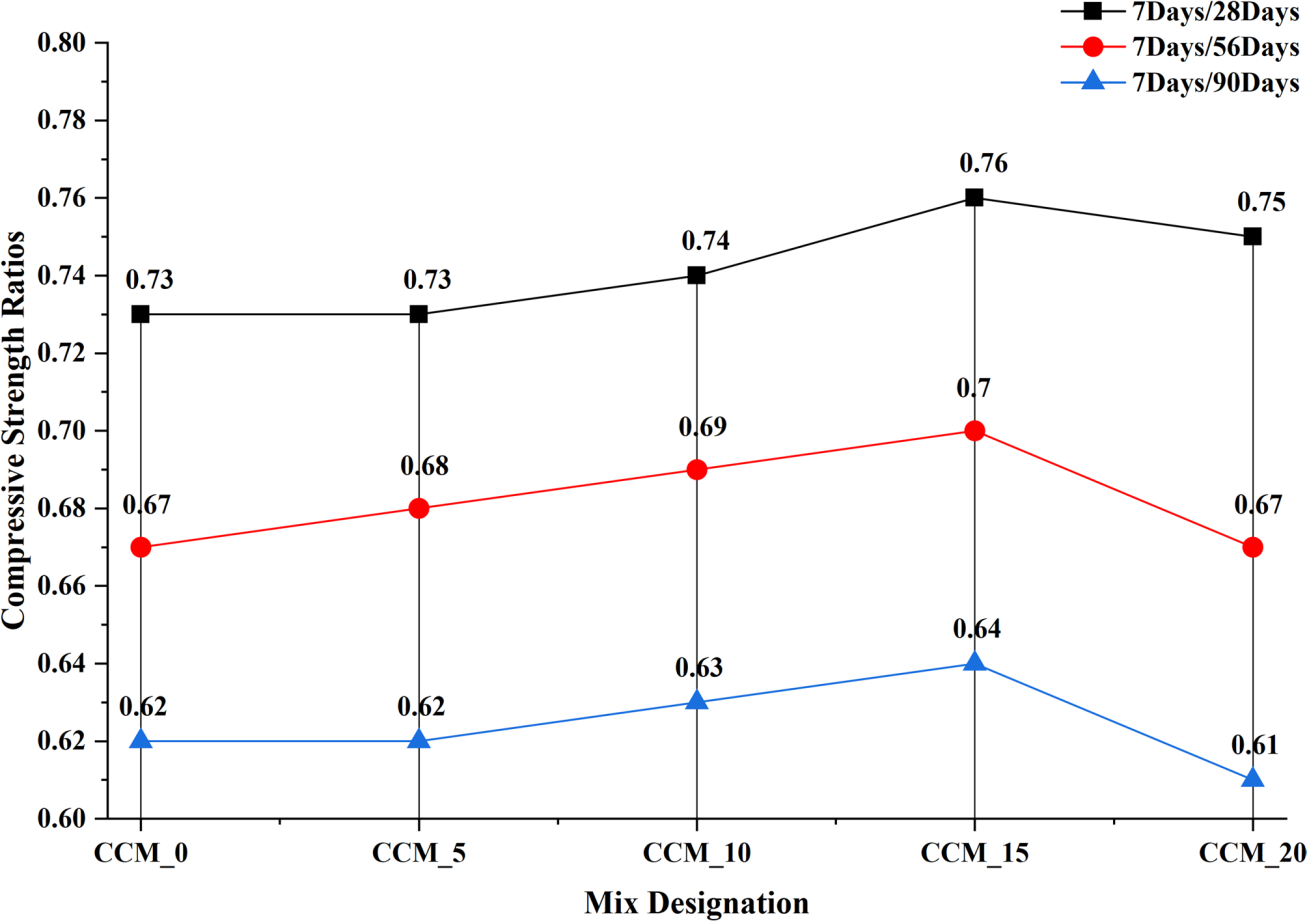
Plot of compressive strength ratios.

**Fig. 11:**
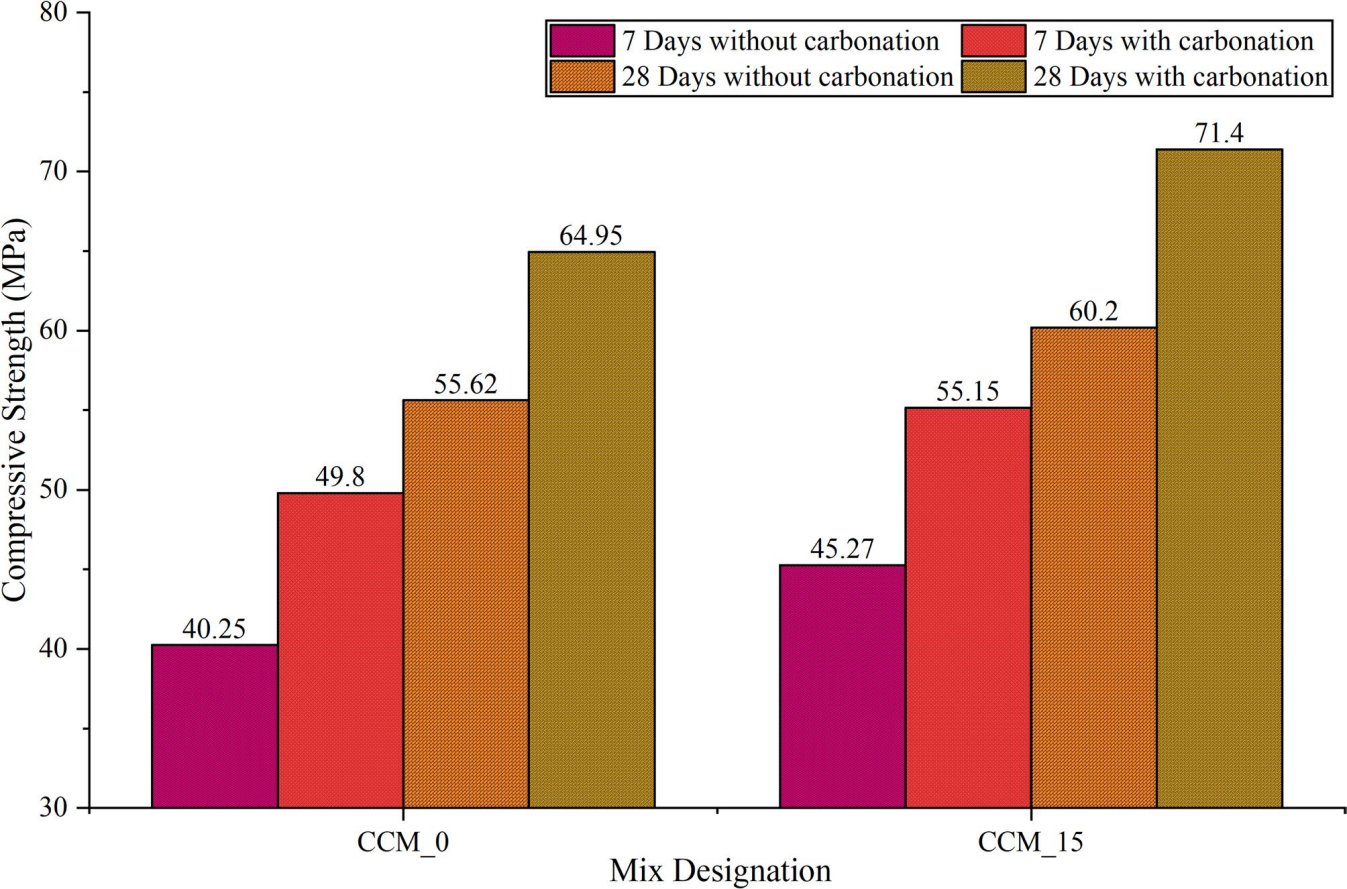
Compressive strength results of calcium carbonate modified concrete of conventional and optimum mix specimens subjected to without carbonation, 7 days of carbonation, and 28 days of carbonation.

**Fig. 12:**
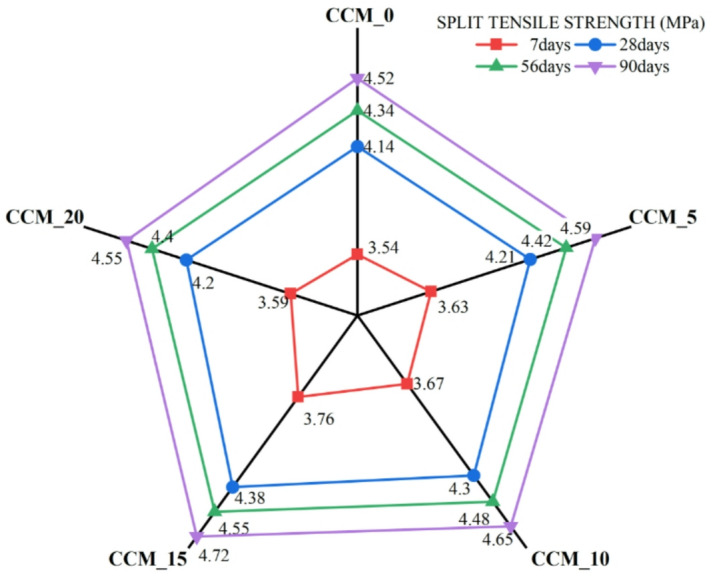
Variation of split tensile strength.

**Fig. 13:**
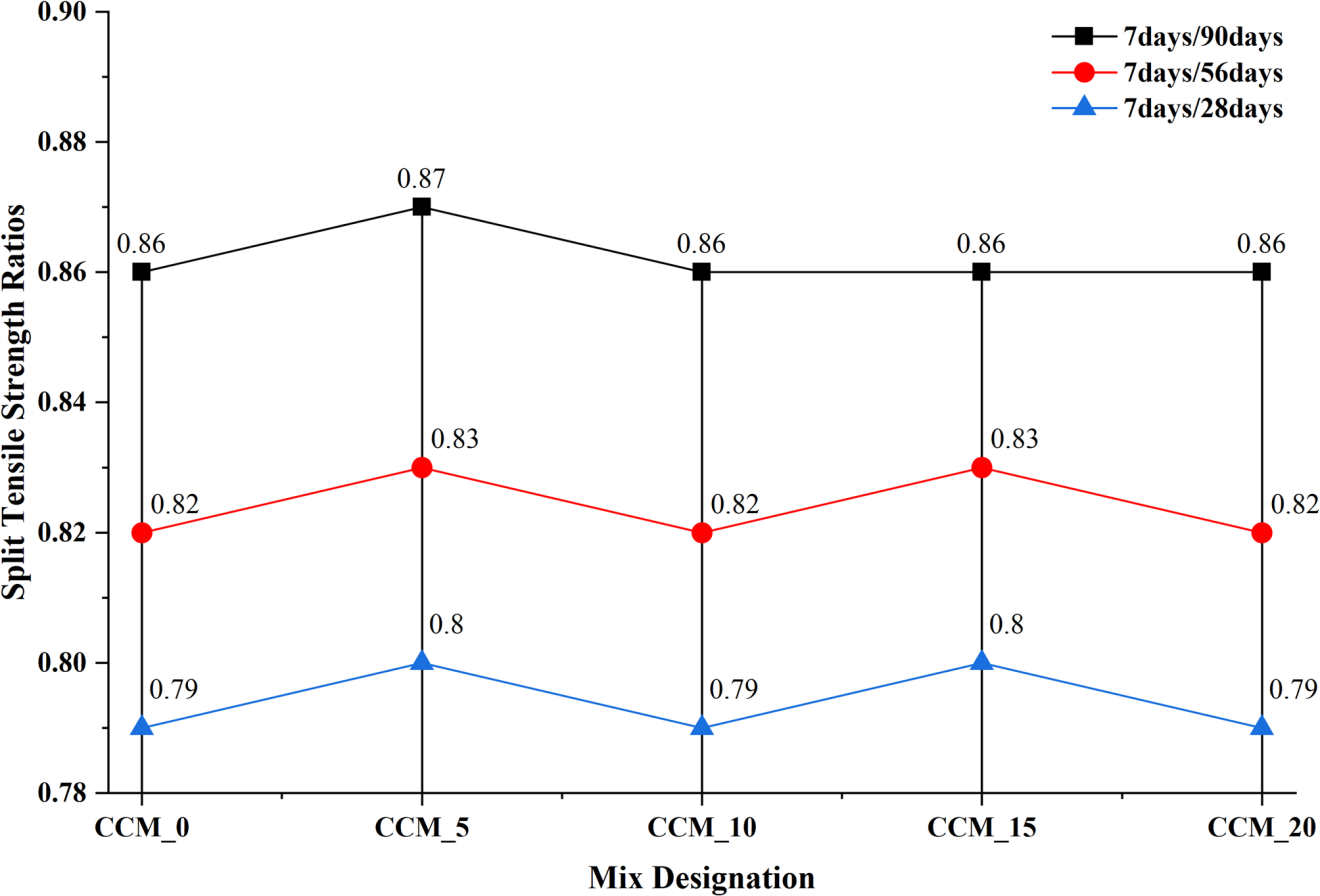
Graph of split tensile ratios.

The effectiveness of CaCO_3_ in modifying the concrete compressive strength at 7, 28, 56 and 90 days was determined according to the IS 516:1959^35^ to determine the effect of CaCO_3_ on mechanical performance. The strength development curves in Fig. 9 indicate that compressive strength continues to increase steadily throughout all the curing periods with the sharpest increase between 7 and 28 days. This enhancement is ascribed to the fact that CaCO_3_ can serve dual roles as a micro filler and nucleation promoter improving early hydration, refining the pore structure, and strengthening interfacial transition zone (ITZ)^33^. CCM_10 and CCM_15 had the best compressive strength at all the times of curing showing that the addition of CaCO_3_ at (10–15) level is the best to pack the mixture, decrease porosity and increase the densification of the matrix. It is at 90 days that both mixes got their highest strengths owing to the unrelenting efforts to refine the hydration products and the microstructure stabilized over time. This behavior can be further confirmed with the help of the strength development ratios (7/28, 7/56 and 7/90 days) as demonstrated in Fig. 10: the mixes CCM10 and CCM15 exhibit the best strength progression, which is reflected in enhanced hydration efficiency and less defects at an early age (microcracking, etc.). Figure 11 shows that carbonated and non-carbonated specimens differ significantly in accelerated carbonation in compressive strength at 7 and 28 days. This is attributed to the fact that the formation of stable CaCO_3_ is achieved by reacting CO_2_ with calcium-bearing hydrates (Ca(OH)_2_ and some of C–S–H), which block pore spaces, decreases the capillary continuity, and raises the density of the matrix^36^. The precipitation of the calcite and the aragonite when a carbonation is present is synergistic with the added CaCO_3_ and the effect results in a more compact microstructure and enhanced strength at a young age. Nevertheless, long-term carbonating can lower the alkalinity and change pore structure meaning the dosage of CaCO_3_ and carbonation environment needs to be well regulated to maximize durability^37^. The findings of the Figs. 11, 12 and 13, in general, indicate that CaCO_3_-modified concrete is able to meet the requirements of mechanical strength, as well as provide considerable sustainability advantages due to the use of an industrial mineral which is abundant. CaCO_3_ improves the packing of the particles, decreases the porosity and speeds up the densification of microstructures, which means that it is a potential constituent of high performance and eco-friendly concrete.

#### Split tensile and flexural strength of CaCO_3_-modified concrete

**Fig. 14:**
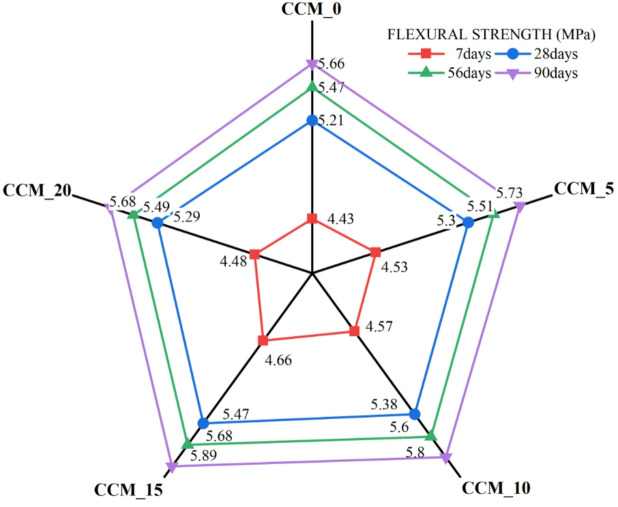
Variation of flexural strength.

**Fig. 15:**
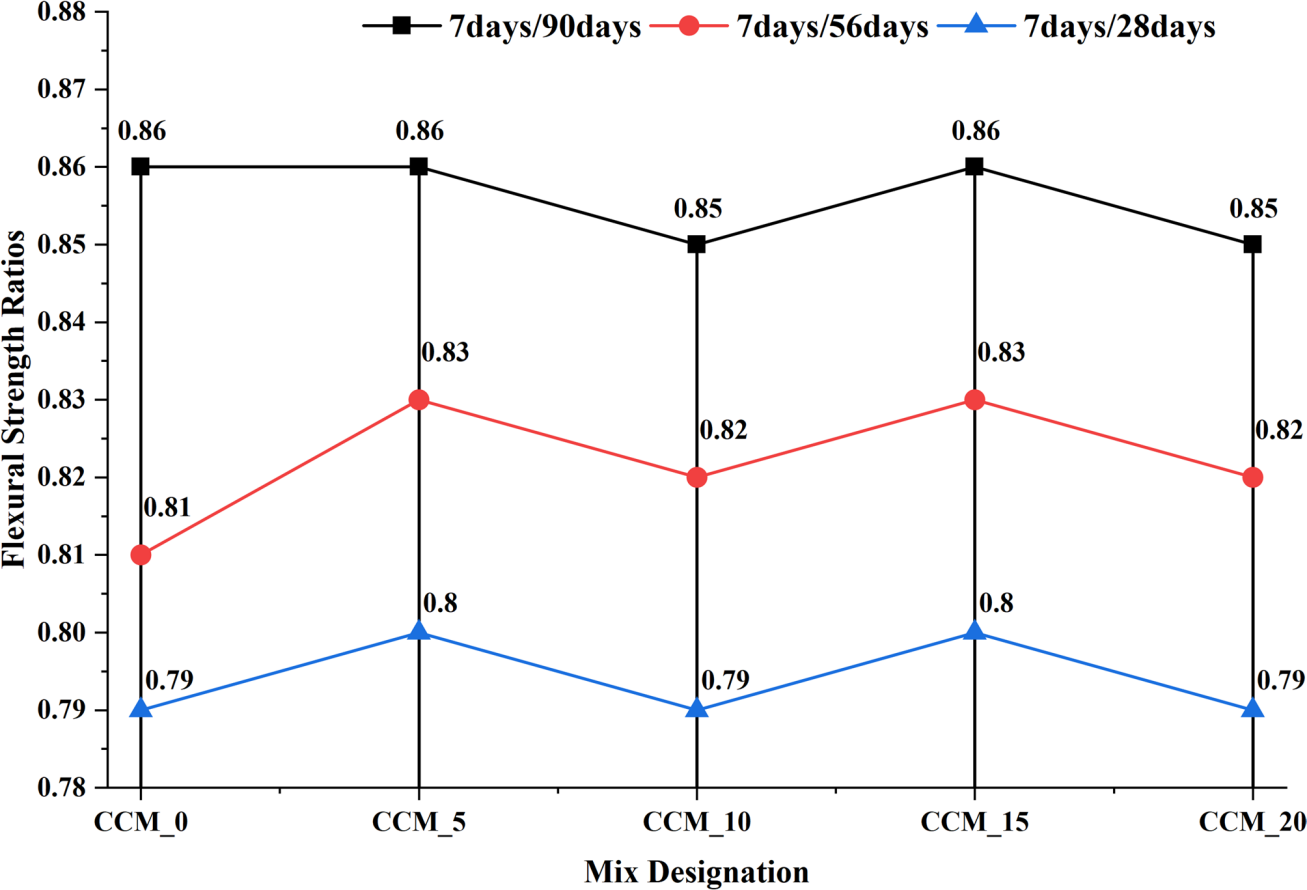
Plot of flexural strength.

To assess the effect of CaCO_3_ on tensile and flexural properties, 7, 28, 56, and 90 days were taken in compliance with IS 516:1959^35^, results of the split tensile strength (Fig. 12) reveal that as the content of CaCO_3_ increased, until 15%, tensile strength and flexural strength also increased steadily. The standard mix had 3.14 MPa at 28 days, which were gradually increasing to 3.54 MPa, 3.63 Mpa and 3.76 Mpa at 5, 10 and 15% CaCO_3_ respectively. The fact that it slightly dropped at 20% (3.59 MPa) suggests that too many fines can lead to agglomeration of particles or less workability. The strengths-development ratios (Fig. 13) also prove that the superior performance of the 15% mix is due to the best stable and progressive increase in strength over curing ages, which is explained by the better nucleation of C–S–H formation, better interfacial transition zone (ITZ) packing and less microcracking^38^. This is also evident in the flexural strength behaviour (Fig. 14) as CaCO_3_ significantly enhanced the load-carrying capacity because of its filler effect, enhanced the densification of the paste, and bridging capacity of micro-cracks. Ordinary concrete registered the following values at 28 days, 5.21 MPa, CaCO_3_-modified mixes registered 5.51 MPa (5%), 5.73 MPa (10%), and a high of 5.89 MPa at 15% replacement. The slight decrease in the 20% (5.49 MPa) indicates that the paste is not as coherent at a higher CaCO_3_ level than its optimal level. This observation is supported by flexural strength ratios (Fig. 15) at all ages of curing because the 15% mix exhibited the largest improvements in flexural strength with better hydration kinetics, more calcium carbonate nucleation sites formed and less pore volume The test results from split tensile and flexural strength assessments demonstrate that 15% CaCO₃ substitution produces the best mechanical results. The enhanced performance results from the combined effects that occur when finely ground CaCO₃ particles function as both filler materials and nucleation sites. The study found that CaCO₃ permitted cementitious materials to achieve better particle packing at moderate application levels because it filled micro-voids present in their matrix structure, which resulted in decreased porosity and enhanced matrix density^39^. The small particles provide additional benefits because they serve as nucleation points, which speed up initial hydration while maintaining even distribution of hydration materials. The improved microstructure of the material increases the strength of ITZ, which connect aggregate and paste materials, thus enhancing their ability to resist cracks and transmit stress when performing tensile and flexural tests. The split tensile and flexural strengths of the material show significant improvement when using this specific replacement level.

In general, split tensile and flexural strength findings prove that 15% CaCO_3_ replacement provides the best combination of microstructural refinement, crack-bridging, and particle packing density, which leads to the enhancement of tensile and flexural resistance^39^. Any replacement levels exceeding this limit will lead to compromised performance in terms of heavy fines, low workability, and even agglomeration. The results highlight the potential of CaCO_3_ as a sustainable cement partial replacement, which improves structural performance and lowers the amount of clinker.

The mechanical performance of the material decreases when the CaCO_3_ content reaches 15% or higher. The introduction of higher replacement levels results in excessive fines which require more water and decrease workability. At higher dosages, the material shows poor dispersion which results in particle agglomeration that creates weak zones and increases potential microcracking. The excessive clinker content dilution leads to decreased availability of reactive phases essential for maintaining strength development. The two effects combined with each other result in reduced tensile strength and lower flexural capacity when multiple substitution levels exist. The split tensile strength values measured at 7, 28, 56, and 90 days in accordance with IS 516:1959 confirm that 15% CaCO_3_ replacement achieves an optimal balance between strength enhancement and workability retention. The environmentally friendly properties of CaCO₃ become apparent when it serves as a partial cement replacement because it decreases clinker usage while enhancing structural performance through microstructural improvements and better stress distribution.

Due to higher hydration degree and tighter microstructure, the strength of conventional concrete is 2.7 Mpa at 28 days, then it increased to 3.0 Mpa, 3.3 Mpa with 5%, 10% and maximum replacement, respectively as illustrated in Fig. 15. There was a slight decrease in the strength to 3.5 Mpa when 20% replacement was carried out; this was probably due to agglomeration of the particles or reduced workability. Due to its improved properties in matrices and fast-hydration, the 15% substitution generated the greatest tensile strength throughout all curing periods with significant strengths between 7 and 28 days demonstrated in Fig. 15.

#### Ultrasonic Pulse Velocity (UPV) and Rebound Hammer (RH) evaluation of CaCO_3_-modified concrete

The effect of incorporation of calcium carbonate and increased carbonation on the quality of concrete was evaluated based on the results of Ultrasonic Pulse Velocity (UPV) and Rebound Hammer (RH) tests, which followed the IS 516 (Part 5/Sec  1):1992 37and IS 13311 (Part 2):1992^38^ respectively. The results of the UVP (Fig. 18) show a definite increase in pulse velocity along with an increase in the content of CaCO_3_ especially after 6 h of carbonation. The CCM_15 mixture recorded the maximum speed at 4589 m/s, with a value of 4499 m/s in the non-carbonated form and thus, falls under the excellence quality (> 4.5 km/s) category. This improvement is mostly due to the micro-filler effect of CaCO_3_, which decreases the size of the pore as well as elevates the stiffness of the cementitious matrix. Carbonation also enhances the microstructure by transforming Ca(OH)_2_ into stable CaCO_3_ polymorphs which seal the micro-voids and enhance the interfacial transition zone (ITZ) this raises the elastic modulus and wave propagation efficiency^39^. The uniformity of the addition of CaCO_3_ and carbonation across mixes is an assertion that the two processes have a synergistic effect on internal homogeneity and structural integrity.

Equally, RH test results (Fig. 17) indicate higher numbers of rebounding at all levels of replacement of carbonated specimens, and again CCM_15 was the strongest in terms of responding to strength. The rebound number was 34, which changed to 36 following a 6-h carbonation, which showed a great change in the surface hardness. This is associated with the densification caused by carbonation at the near-surface area whereby surface stiffness due to the formation of finely dispersed crystals of CaCO_3_ and microcracking is minimized. CaCO_3_ particles also enhance better surface packing and paste continuity which further increases values of hardness. RH is only an approximate estimate of compressive strength but the trends are highly correlated with UPV, which validates the fact that surface and internal densification process takes place simultaneously during CaCO_3_ modification and carbonation.

**Fig. 16:**
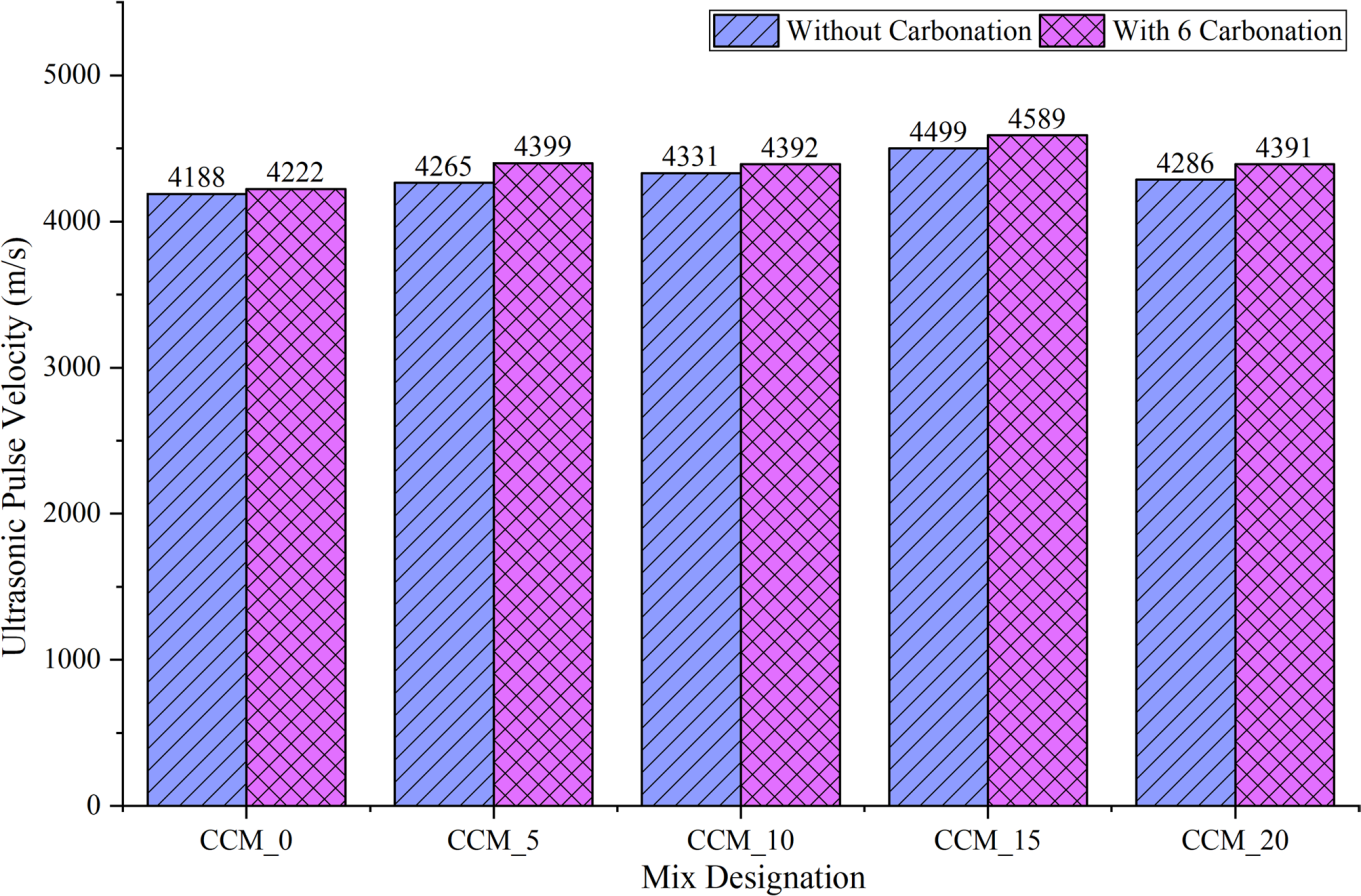
UPV test results on concrete without and with 6 h of carbonation.

**Fig. 17:**
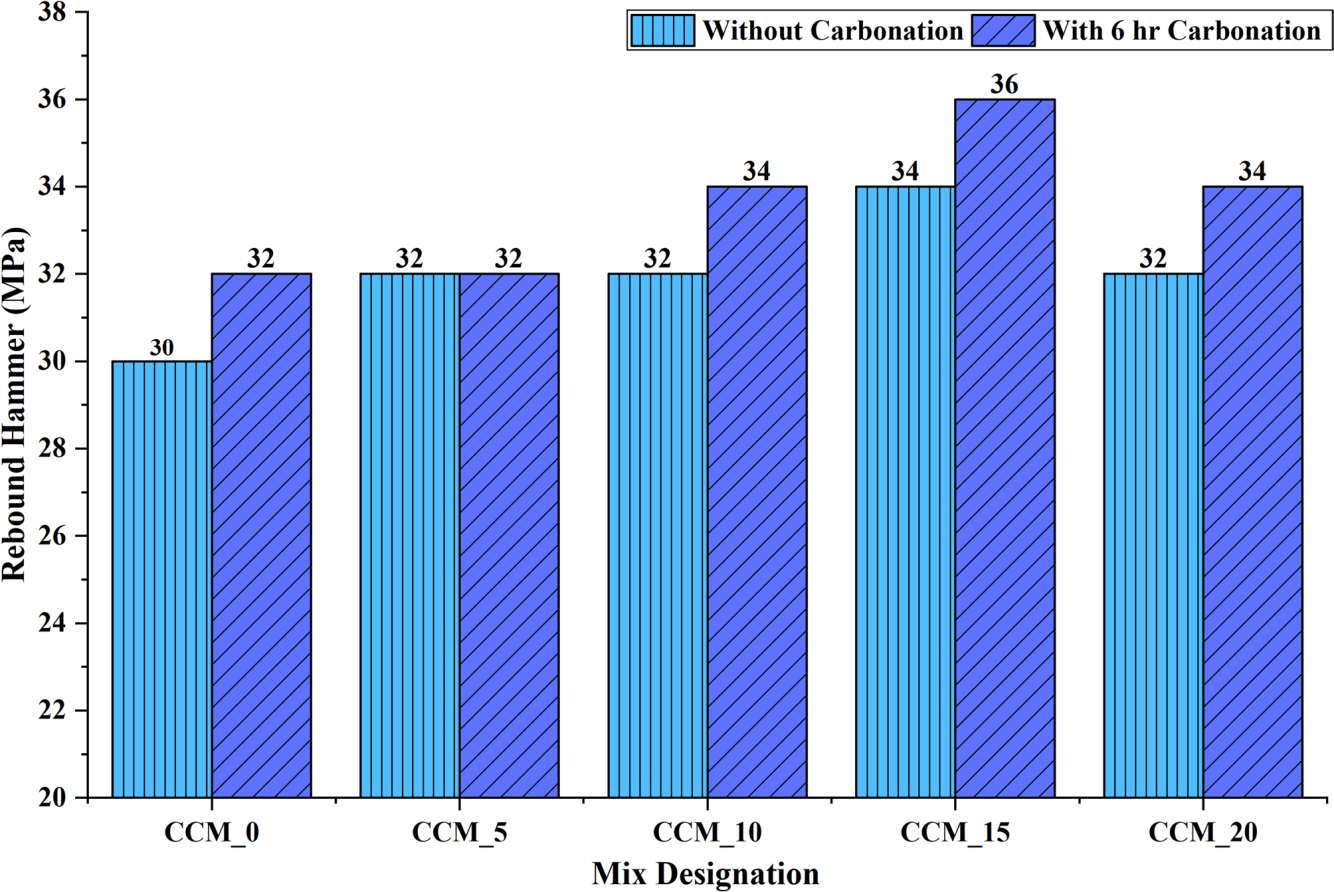
Rebound hammer test results on concrete without and with 6 h of carbonation.

Altogether, the equivalent results of the UPV and RH indicate that the 15% replacement of CaCO_3_ with help of controlled carbonation results in the best changes in the internal compactness, surface hardness and mechanical performance. The consistent patterns in Figs. 16 and 17 confirm the use of CaCO_3_ in the construction industry as an efficient micro-filler and nucleation promoter, whereas carbonation serves as a secondary densification, and CaCO_3_ modified concrete is a promising route towards high-performance, durable, and low-carbon construction materials.

#### Microstructural analysis of CaCO_3_-modified concrete

The high-resolution Field Emission Scanning Electron Microscopy (FESEM) was used to analyze the microstructural development of CaCO_3_-modified concrete (CM). Figure 20a of SEM images shows that there is a considerable disparity in the microstructure of carbonated and non-carbonated concrete^39^. The concrete without the incorporation of CaCO_3_ (CCM_0), had a porous matrix, which was typified by microcracks and non-reacted cement particles, thus, resulting to a loosely packed C-S-H phase, enhancing permeability and lowering durability^40^. Such microstructural defects are the main reasons behind the lesser compressive, split tensile, and flexural strengths of the conventional mix since the imperfections in the ITZ (interfacial transition zone) compromise the load-carrying ability of the concrete.

**Fig. 18:**
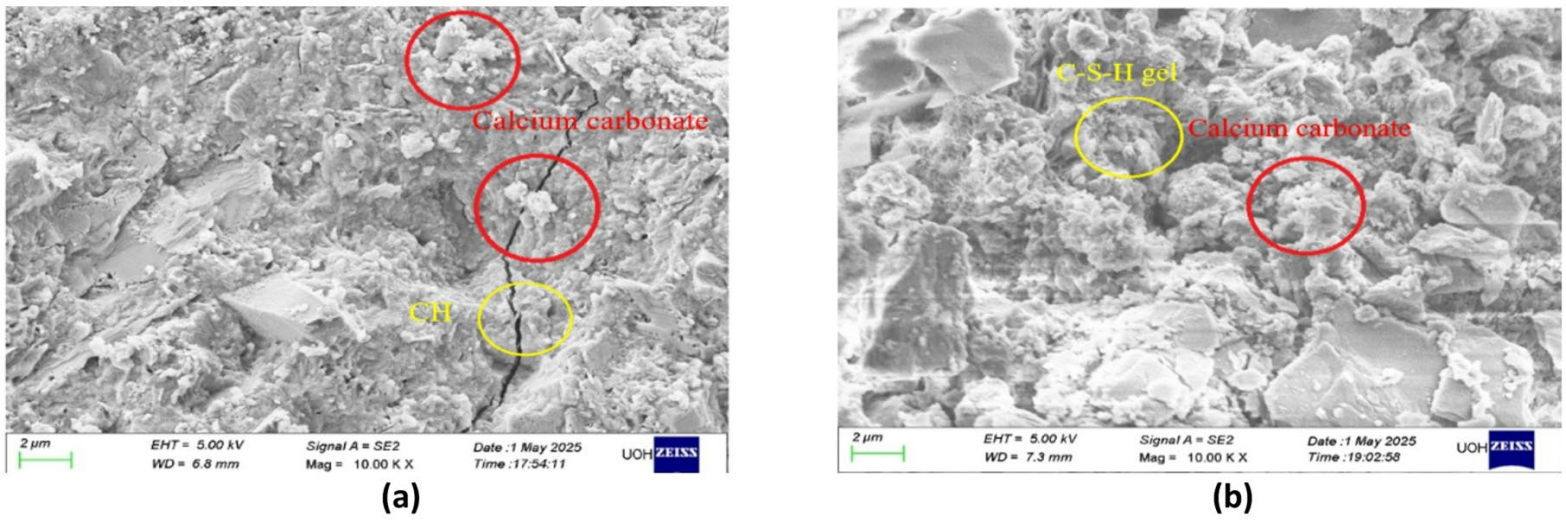
Scanning electron microscopy of concrete (**A**) Optimum without carbonation and (**B**) Optimum with carbonation.

The CaCO_3_-modified concrete (CCM_5, CCM_10, CCM_15, CCM_20) exhibits, however, significantly denser morphologies than the control mix. This is made possible by the filler effect of CaCO_3_ that is effective in reducing porosity and enhanced gap-filling by the concrete matrix filling. The figures of Fig. 18a demonstrate that CaCO_3_ particle (red circles) fills the vacuity of C–S–H gel (yellow circles) and cement particles, enhancing densification of the microstructure. Such densification was more pronounced with the mixes containing 10–15% CaCO_3_ replacement in which a balance between workability, durability and mechanical performance was realized. The carbonation process also produced the formation of CaCO_3_ crystals that could be observed in the carbonated specimens where the calcium hydroxide (Ca(OH)_2_) reacted with the available CO_2_ in the air and the resulting CaCO_3_ formed was stable^41^. This also seals microcracks, increases the interlocking of C–S–H phases, and to a large extent strengthens the compressive strength due to the reduction in the space between voids in the matrix.

The SEM analysis presented in Fig. 18b identifies the role played by the densification and ensuing carbonation process triggered by the CaCO_3_ to enhance the long-term viability and high compressive strength of the modified concrete^42^. The increase in compressive strength directly depends on the enhanced particle packing and reduced material porosity because void spaces in the material decrease, which leads to greater material stiffness. The microstructure with higher density shows superior tensile and flexural strength because the split tensile and flexural strength tests show that 15% CaCO_3_ replacement produced better results than the other two strength tests. The increase in compressive strength results from better particle packing and lower porosity because these factors lead to reduced void space and enhanced continuity of the material. A denser microstructure enables better stress distribution, which results in superior tensile and flexural strength. Experimental results indicate that 15% CaCO_3_ replacement provides the optimal balance, which yields the highest values across compressive, split tensile, and flexural strength tests. Carbonation improves performance because it causes CaCO_3_ to form in pore spaces, which improves microstructural quality and strengthens interfacial bonds. The protection of reinforcement will be diminished because excessive carbonation decreases alkalinity. Microstructural observations support that a 10–15% CaCO_3_ replacement level achieves a favorable balance between workability matrix densification and mechanical enhancement, which matches the measured strength results^43^.

The scale bar in the SEM pictures, which was set at 2 μm, also gives more details on the fine-scale characteristics that affect the entire concrete behavior. One can directly relate the presence of nano-sized CaCO_3_ particles along with their impact on enhancing pore structure to the increase in mechanical strengths, i.e. compressive, split tensile, and flexural strengths. Such results highlight that CaCO_3_ is a promising partial replacement material, which can increase the strength and sustainability of concrete^39^.

#### Electrochemical impedance spectroscopy (EIS)

**Fig. 19:**
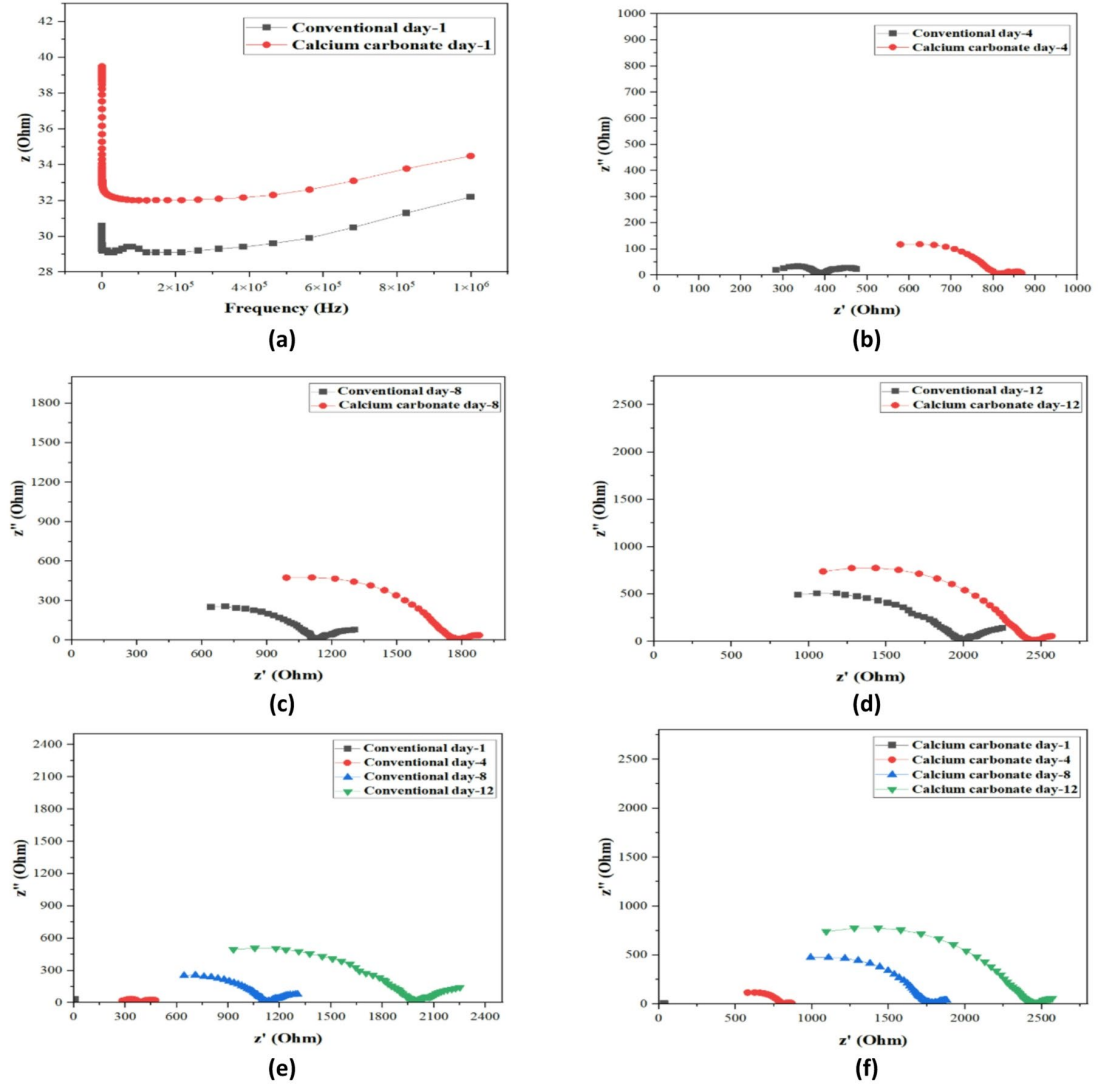
Nyquist plot for Conventional and CM concrete at (**a**) day 1, (**b**) 4 days, (**c**) 8 days, (**d**) 12 days, (**e**) Conventional concrete, and (**f**) Calcium Carbonate Modified Concrete.

**Fig. 20:**
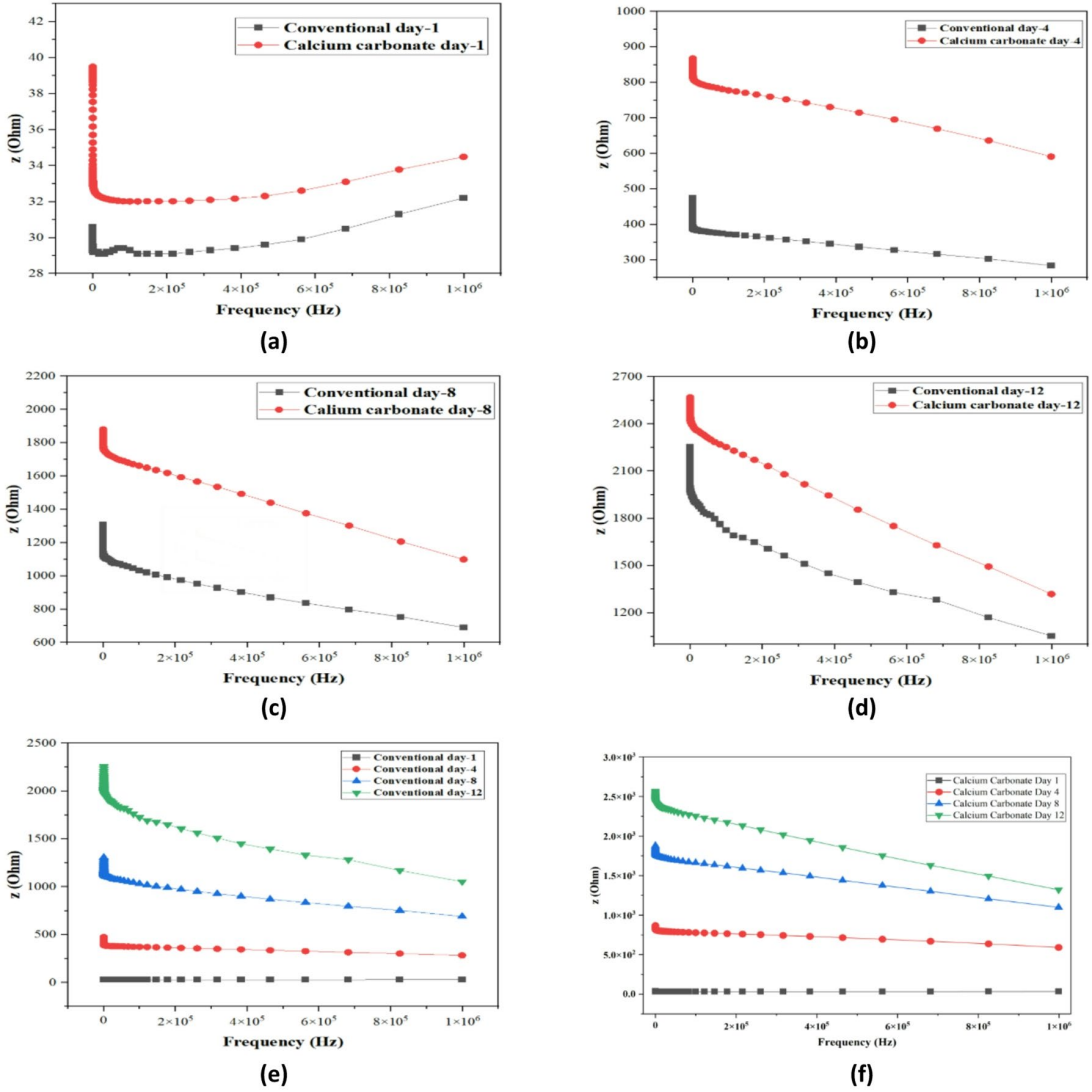
Bode’s plot for conventional and CM concrete at (**a**) day 1, (**b**) 4 days, (**c**) 8 days, (**d**) 12 days, (**e**) Conventional concrete comparison, and (**f**) Calcium carbonate modified concrete comparison.

Electrochemical Impedance Spectroscopy (EIS) is an elaborate approach that can give a deeper investigation of the electrochemical behavior of concrete through the measurement of the impedance response at different frequencies, which can deliver information on how structure changes throughout the process of concrete and its stability. EIS was used in the present study in the conventional and the calcium carbonate (CaCO_3_)-modified concrete samples within a 12-day period. The Nyquist plots (Figs. 19 and 20) are the figures that show real and imaginary impedance (Z′ and Z′ respectively) versus the hydration time and indicate the key information about the hydration kinetics and pore structure and interfacial properties^44^.

Figure 19a–f demonstrates the Nyquist plot of standard and modified with CaCO_3_ concrete specimens of various curing days. In the normal concrete (Fig. 19e), the impedance response gradually improved as time passed and the curing process, which had a clear arc. At day 1, the impedance was small because of large volumes of water-filled pores that enhanced electrical conductivity as observed in Fig. 19a. At day 4 (Fig. 19b) the impedance became large which indicates that the process of hydration was initiated and formation of hydration products took place. The same tendency was observed on days 8 and 12 (Fig. 19c,d) when the impedance increased to a higher value, which was associated with the more compact microstructure and lower porosity with the hydration products.

Conversely, Nyquist plots of the CaCO_3_-modified concrete (Fig. 21f) exhibit a varying pattern. Figure 19a (day 1) shows that the impedance of CaCO_3_-modified concrete was a bit greater than conventional concrete, which means that the solution of pore conductivity was more conductive because the fine particles of CaCO_3_ required more water. This implies that at the beginning, the hydration of the CaCO_3_ needs a higher amount of water but with time, the filler effect of CaCO_3_ is more pronounced. The carbonation reaction causes the CaCO3 to react with CO_2_ into stable calcium carbonate precipitates, which fill in the voids and enhance the microstructural density as indicated by the overlaying impedance on day 4 (Fig. 19b), and subsequent increase at day 8 (Fig. 19c) and day 12 (Fig. 19d).

The further explanation of the behavior of both conventional and CaCO_3_ modified concrete is presented in the Bode plots as illustrated in Fig. 20a–f. Impedance magnitude at low frequencies (e.g.0.1 Hz) was lower in the case of CaCO_3_-modified concrete which means it has better conductivity compared to the conventional mix. This is explained by the fact that the carbonation of CaCO_3_ results in a denser matrix that maximizes the pore structure and enhances the conduction pathway. At day 12, the magnitude of the impedance of CaCO_3_-modified concrete (Fig. 20e,f) was smaller than in the case of conventional concrete, which is indicative of a more conductive microstructure that allows increased ion transport and improved performance^45^. The phase angle changed also to the lower values in the mid-to-low frequencies implying a resistive response and less porosity which is a good attribute towards durability.

The results of electrochemical impedance spectroscopy (EIS), which originated from Nyquist and Bode plots, demonstrate that CaCO_3_ addition speeds up hydration processes while producing stable carbonation products. The improved ionic transport behavior results from reduced charge transfer resistance and new impedance characteristics that emerged from the refined pore structure. The process of carbonate precipitation fills pores and compacts the matrix, which results in improved mechanical strength and durability. The CaCO_3_ functions as a supplementary material because it enhances both electrochemical performance and structural properties while enabling more sustainable concrete production through its use as a clinker replacement material.

### Comparison of strength and cement content studies for M30 grade of concrete

Table 5. is a comparative analysis of cement content, grade of concrete, supplementary cementitious materials (SCMs) used and the compressive strength at 28 days achieved in different studies. The comparison reveals the effect of various SCMs such as crumb rubber, micro-silica, biomass ash, fly ash, silica fume, rice husk ash (RHA), GGBS and calcium carbonate (CaCO_3_) on the mechanical characteristics of M30 grade concrete. The present work (row 8) shows that CaCO3 replacement of cement (15 per cent) increased significantly compressive strength of M30 grade concrete to 60.2 MPa at 28days. This is quite a high value than the strengths that are reported of other SCMs. Besides enhancing strength, CaCO_3_ addition is also part of the sustainability, as it provides less content of cement in the final product, and decreases the CO_2_ emissions generated during cement production^46^. The results highlight the suitability of CaCO_3_ in improving the performance of concrete and at the same time minimizing the environmental impact of construction materials.

**Table 5:** Strength and cement content study comparison for M30 grade of concrete.

S. No.	Cement (kg/m^3^)	Grade of concrete	SCM’s used & substitution on level (%) in cement	Compressive strength (MPa) at 28 days	References
1	400	M30	Crumb rubber + micro-silica; 5–20% crumb rubber (various mixes)	29	^47^
2	427	M30	BMIA (incinerated biomass ash) 5,10,15,20% replacement of cement	36.54	^48^
3	262.5	M30	Various SCMs (fly ash, silica fume, RHA) — up to 20%	34	^49^
4	331.5	M30	Silica fume and Rice Husk Ash (RHA), 5–20%	45	^50^
5	128	M30	GGBS (10–25%)	38.5	^51^
6	320	M30	Laterite (15%)	42.14	^52^
7	301.7	M30	High volume GGBS (15–20%)	32.4	^53^
8	190	M30	Calcium Carbonate (15%)	60.2	Present study

## Conclusions

This paper discussed the use of calcium carbonate (CaCO_3_) as a partial substitute of cement in concrete in order to improve its mechanical properties, durability, and sustainability. The experimental assessment involved a series of tests on concrete mixes of varying levels of replacement of CaCO_3_ (5, 10, 15, and 20 per cent), which were assessed on the basis of compressive strength, split tensile strength, flexural strength, non-destructive testing (NDT), and microstructural analysis. The findings were evaluated to see the best content of CaCO_3_ that will give a balance between the performance and environmental gains.

- The CaCO_3_ 10% (CCM_10) and 20% (CCM_20) mixes were found to have the highest initial strength gains and lower shrinkage cracking, and 15% replacement of CaCO_3_ (CCM_15) showed the best overall performance between strength and thus attained 3.6 MPa split tensile strength in 28 days; this was better than the conventional concrete.
- Replacement of 20% reduced strength to some extent as a result of particle agglomeration and lower workability which indicates that control of CaCO_3_ dosage was important to achieve good performance.
- The UPV and Rebound Hammer tests showed that CCM_15 with 6 h carbonation had the optimal quality and hardness of the surface and this implied that there was limited porosity and high densification of the concrete.
- The FeSEM analysis showed the presence of a more dense microstructure with reduced pores and cracks in the CaCO_3_-modified concrete which is further increased by the precipitation of CaCO_3_ crystals due to carbonation.
- The EIS analysis showed that early hydration was slower in the CaCO_3_ mixes, but as time went on, the microstructure became refined and hence the better long-term durability and carbonation resistance.

The amount of replacement of the CaCO_3_ (15%) was determined to be the most effective amount of CaCO_3_ that improves the mechanical, durability, and sustainability of the concrete. Such replacement was effective in strengthening as well as helped in environmental sustainability by reducing cement makeup and minimizing CO_2_ emissions. The results prove the effectiveness of CaCO_3_-modified concrete as a more environmentally friendly, high-performance building material that has many long-term advantages both in terms of strength and durability.

## Data Availability

The datasets used and/or analysed during the current study are available from the corresponding author on reasonable request.

## References

[1] Alawi Al-Naghi, A. A., Ahmad, A., Amin, M. N., Algassem, O. & Alnawmasi, N. Sustainable optimisation of GGBS-based concrete: De-risking mix design through predictive machine learning models. *Case Stud. Constr. Mater.* **23**, e04900 (2025).

[2] Ma, Z., Shen, J., Wang, C. & Wu, H. Characterization of sustainable mortar containing high-quality recycled manufactured sand crushed from recycled coarse aggregate. *Cem. Concr Compos.* **132**, 104629 (2022).

[3] Ma, Z., Wang, B., Zhang, Z., Zhang, Y. & Wang, C. New insights into the effects of silicate modulus, alkali content and modification on multi-properties of recycled brick powder-based geopolymer. *J. Build. Eng.* **97**, 110989 (2024).

[4] Wu, H., Liu, X., Wang, C., Zhang, Y. & Ma, Z. Micro-properties and mechanical behavior of high-ductility engineered geopolymer composites (EGC) with recycled concrete and paste powder as green precursor. *Cem. Concr Compos.* **152**, 105672 (2024).

[5] Wang, C., Zhang, Z., Liu, X., Zhang, Y. & Ma, Z. Elucidating the role of recycled concrete aggregate in ductile engineered geopolymer composites: Effects of recycled concrete aggregate content and size. *J. Build. Eng.* **95**, 110150 (2024).

[6] Sharma, R., Jang, J. G. & Bansal, P. P. A comprehensive review on effects of mineral admixtures and fibers on engineering properties of ultra-high-performance concrete. *J. Build. Eng.* **45**, 103314 (2022).

[7] Singh, R. P. et al. Fly ash, GGBS, and silica fume based geopolymer concrete with recycled aggregates: Properties and environmental impacts. *Constr. Build. Mater.* **378**, 131168 (2023).

[8] Bavithra, K. & Mohana, R. Sustainable development of durable and novel nano GGBS impregnated eco-friendly green concrete using micro structural characterization and techno-economic sustainability analysis. *Constr. Build. Mater.* **470**, 140565 (2025).

[9] Saingam, P. et al. Synergizing Portland Cement, high-volume fly ash and calcined calcium carbonate in producing self-compacting concrete: A comprehensive investigation of rheological, mechanical, and microstructural properties. *Case Stud. Constr. Mater.* **21**, e03832 (2024).

[10] Jin, F., Zhao, M., Xu, M. & Mo, L. Maximising the benefits of calcium carbonate in sustainable cements: Opportunities and challenges associated with alkaline waste carbonation. *NPJ Mater. Sustain* **2**, (2024).

[11] Chang, C. et al. Enhancing mechanical properties of high-strength recycled concrete with basalt fiber and nano-calcium carbonate: Experimental and numerical investigations. *Constr. Build. Mater.* **489**, 142264 (2025).

[12] Compaore, A. et al. Performance of foam concrete containing low-temperature calcined clay as a partial replacement of Portland cement. *J. Mater. Res. Technol.* **38**, 1761–1781 (2025).

[13] Abellan-Garcia, J., Iqbal Khan, M., Abbas, Y. M., Martínez-Lirón, V. & Carvajal-Muñoz, J. S. The drying shrinkage response of recycled-waste-glass-powder-and calcium-carbonate-based ultrahigh-performance concrete. *Constr. Build. Mater.* **379**, 131163 (2023).

[14] Bawab, J., El-Hassan, H., El-Dieb, A., Khatib, J. & El-Mir, A. Utilization of calcium carbide residue as a concrete component: A comprehensive review. *Case Stud. Constr. Mater.* **22**, e04823 (2025).

[15] Shah, H. A., Wang, Y., Banthia, N. & Meng, W. Enhancing nano-CaCO3 dispersion with cellulose nanocrystals for high-strength low-carbon concrete. *Cem. Concr Compos.* **164**, 106227 (2025).

[16] Harish, K., Madupu, L. N. K. S. & Satyanarayana, S. V. An experimental study on strength of concrete having calcium carbonate as a partial replacement material to cement and natural river sand. *Res. Eng. Struct. Mater.* **11**, 697–712 (2024).

[17] Sua-iam, G. & Jamnam, S. Influence of calcium carbonate on green self-compacting concrete incorporating porcelain tile waste as coarse aggregate replacement. *Case Stud. Constr. Mater.* **19**, e02366 (2023).

[18] Hefni, M. & Ali, M. A. The potential to replace cement with nano-calcium carbonate and natural pozzolans in cemented mine backfill. *Adv. Civ. Eng.* **2021**, 5574761 (2021).

[19] Hargis, C. W. et al. Microstructure development of calcium carbonate cement through polymorphic transformations. *Cem. Concr. Compos.* **153**, 105715 (2024).

[20] Rathna Chary, M. et al. The properties of geo-polymer concrete by partial replacement of cement with GGBS & fly ash. *MATEC Web Conf.* **392**, 01006 (2024).

[21] IS 269–2015. | PDF | Cement | Chemical Substances. Reaffirmed (2020). https://www.scribd.com/document/690243322/IS-269-2015-Reaffirmed-2020

[22] of Indian Standards, B. IS 2720-3-1. Methods of test for soils, Part 3: Determination of specific gravity, Section 1: Fine grained soils. (1980).

[23] Ahmad, J. et al. A comprehensive review on the Ground Granulated Blast Furnace Slag (GGBS) in concrete production. *Sustain. 2022*. **14**, 8783 (2022).

[24] Siddique, R. Utilization (recycling) of iron and steel industry by-product (GGBS) in concrete: Strength and durability properties. *J. Mater. Cycles Waste Manag*. **163** (16), 460–467 (2013). (2013).

[25] Meimaroglou, N. & Mouzakis, C. Evaluation of the effects of intrinsic soil calcium carbonate and iron oxides on the properties of earthen building materials by means of engineered soils. *Low-carbon Mater. Green Constr.* **3**, 8- (2025).

[26] Lertwattanaruk, P., Sua-iam, G. & Makul, N. Effects of calcium carbonate powder on the fresh and hardened properties of self-consolidating concrete incorporating untreated rice husk ash. *J. Clean. Prod.* **172**, 3265–3278 (2018).

[27] Yu, Y. & Guoqing, G. Quantification of GGBS hydration using deep learning—A comparison with SEM-EDS mapping, PONKCS XRD and isothermal calorimetry methods. *Cem. Concr Res.* **197**, 107960 (2025).

[28] Song, X. et al. Effects of K ions on the vaterite CaCO3 formation using the steamed ammonia liquid waste as calcium sources. *Powder Technol.* **463**, 121172 (2025).

[29] Amal Mohammed, A. M. et al. Influence of nano CaCO3 on pore structure and compressive strength in concrete bricks. *J. Build. Eng.* **105**, 112520 (2025).

[30] Bonavetti, V. L., Rahhal, V. F. & Irassar, E. F. Studies on the carboaluminate formation in limestone filler-blended cements. *Cem. Concr Res.* **31**, 853–859 (2001).

[31] Mehdizadeh, H. & Hajmohammadian Baghban, M. Effect of sea salt coupled with reaction temperature on aqueous carbonation of recycled concrete powder: Insights into CO2 sequestration and CaCO3 polymorphism. *Case Stud. Constr. Mater.* **23**, e05521 (2025).

[32] Kliková, K., Holeček, P., Koňáková, D., Stiborová, H. & Nežerka, V. Exploiting *Bacillus pseudofirmus* and *Bacillus pseudofirmus* to promote CaCO3 and AFt phase formation for stabilizing waste concrete fines. *Cem. Concr Compos.* **155**, 105839 (2025).

[33] Zhang, R., Srivastava, M. G., Braem, A., Mignon, A. & Wang, J. Mesoporous silica nanoparticles loaded urea for enhancement of the cohesion of biogenic CaCO3 and its adhesion with recycled concrete aggregates. *J. Build. Eng.* **99**, 111528 (2025).

[34] Shah, H. A., Du, J. & Meng, W. Low-carbon UHPC with carbonated blast furnace slag: Impact of mineral composition, carbonation degree, and CaCO3 polymorphs. *Cem. Concr Compos.* **160**, 106039 (2025).

[35] Shah, H. A. & Meng, W. Improving the mechanical properties of cement paste with carbonated blast furnace slag by tailoring CaCO3 polymorphs and increasing carbonation degree. *Cem. Concr Compos.* **165**, 106343 (2026).

[36] Neelamegam, P. & Muthusubramanian, B. Influence of Polyethylenimine (PEI) in enhancement of microstructure and surface morphology of recycled construction and demolition waste aggregate in concrete by carbonation. *Constr. Build. Mater.* **405**, (2023).

[37] Hartono, J., Ekaputri, J. J. & Purwanto & Effects of HVFA with the addition of bottom ash, NaOH, and CaCO3 on self-compacting concrete (SCC) in tidal environments. *Case Stud. Constr. Mater.* **22**, e04593 (2025).

[38] Zheng, X. et al. Experimental investigation and mesoscale simulation of frost resistance of low-heat cement mortar modified with nano-CaCO3. *Constr. Build. Mater.* **495**, 143616 (2025).

[39] Wang, Z. et al. Effect of CO_2_-absorbed CaCO_3_ on the strength of blast furnace slag cement mortar. *Constr. Build. Mater.* **496**, 143744 (2025).

[40] Neelamegam, P. & Muthusubramanian, B. Evaluating embodied energy, carbon impact, and predictive precision through machine learning for pavers manufactured with treated recycled construction and demolition waste aggregate. *Environ. Res.* **248**, 118296 (2024). 38280525 10.1016/j.envres.2024.118296

[41] Zhu, H. et al. Investigation on the effect of nano-CaCO_3_/hydromagnesite and properties of lamellar hydrates bonded alumina-magnesia refractory concrete. *Constr. Build. Mater.* **458**, 139561 (2025).

[42] Wang, T., Fan, X., Gao, C. & Qu, C. Macro-mechanics and microstructure of nanomaterial-modified geopolymer concrete: A comprehensive review. *J. Wuhan Univ. Technol. Sci. Ed.* **40**, 204–214 (2025).

[43] Guan, S. et al. Non-equilibrium molecular dynamics simulation of CaCO_3_ nucleation and growth in C-S-H gel pores under various loading conditions. *Colloids Surf. Physicochem. Eng. Asp*. **713**, 136499 (2025).

[44] Kumar, B. N., Sunil, B. S. & Bhat, P. K. Exploring the synergistic effects of quartz powder and GGBS in sustainable standard strength concrete: insights from carbonation and electrochemical impedance spectroscopy (EIS). *Discov. Mater.* **5**, 147 (2025).

[45] Zhao, K. et al. Effect of CO2 mixing on the rheological and electrochemical properties of fresh mortar at the early age. *Cem. Concr. Compos.* **155**, 105817 (2025).

[46] Lyu, H. J., Yu, J., Jeon, D. & Oh, J. E. CaCO3 dissolution-driven enhancement of strength and microstructure in clinker-free CaCO3-blended GGBFS binder via hydrated Al2(SO4)3. *Constr. Build. Mater.* **458**, 139762 (2025).

[47] Filippova, I. V., Fekry, A. M., Medany, S. S. & Filippov, L. O. The interaction mechanism of dolomite mineral and phosphoric acid and its impact on the Ti alloy corrosion. *Sci. Rep.* **15**, 1–23 (2025). 39922829 10.1038/s41598-025-87514-6 PMC11807172

[48] Parvan, M. G., Voicu, G., Badanoiu, A. I., Nicoara, A. I. & Vasile, E. CO2 sequestration in the production of Portland cement mortars with calcium carbonate additions. *Nanomater 2021*. **11**, 875 (2021). 10.3390/nano11040875 PMC8066617 33808156

[49] He, Z., Shao, X. & Chen, X. Effect of carbonation treatment on the strength and CO2 uptake rate of composite cementitious material with a high steel slag powder content. *Mater.*. **16**, 6204 (2023). 10.3390/ma16186204 PMC10532887 37763482

[50] Li, X. & Cao, M. Recent developments on the effects of micro- and nano-limestone on the hydration process, products, and kinetics of cement. *Mater.*. **17**, 2133 (2024). 10.3390/ma17092133 PMC11084965 38730939

[51] Poudyal, L., Adhikari, K. & Won, M. Nano calcium carbonate (CaCO3) as a reliable, durable, and environment-friendly alternative to diminishing fly ash. *Mater.* **14**, 3729 (2021). 10.3390/ma14133729 PMC8269888 34279297

[52] Yang, H., Che, Y. & Leng, F. High volume fly ash mortar containing nano-calcium carbonate as a sustainable cementitious material: Microstructure and strength development. *Sci. Rep.* **8**, 16439 (2018). 10.1038/s41598-018-34851-4 PMC6219606 30401939

[53] Liu, X. et al. High-performance oil well cement with modified calcium carbonate whiskers: Enhancing durability under HTHP conditions. *Mater.*. **18**, 1021 (2025). 10.3390/ma18051021 PMC11901247 40077247

